# Correcting for Genomic Inflation Leads to Loss of Power in Large‐Scale Genome‐Wide Association Study Meta‐Analysis

**DOI:** 10.1002/gepi.70016

**Published:** 2025-08-06

**Authors:** Archit Singh, Lorraine Southam, Konstantinos Hatzikotoulas, Nigel W. Rayner, Ken Suzuki, Henry J. Taylor, Xianyong Yin, Ravi Mandla, Alicia Huerta‐Chagoya, Andrew P. Morris, Eleftheria Zeggini, Ozvan Bocher

**Affiliations:** ^1^ Technical University of Munich (TUM), TUM School of Medicine and Health, Graduate School of Experimental Medicine Munich Germany; ^2^ Institute of Translational Genomics Helmholtz Zentrum München‐ German Research Center for Environmental Health Neuherberg Germany; ^3^ Munich School for Data Science (MUDS) Helmholtz Zentrum München‐ German Research Center for Environmental Health Neuherberg Germany; ^4^ Department of Diabetes and Metabolic Diseases, Graduate School of Medicine University of Tokyo Tokyo Japan; ^5^ Department of Statistical Genetics Osaka University Graduate School of Medicine Suita Japan; ^6^ Center for Precision Health Research, National Human Genome Research Institute National Institutes of Health Bethesda Maryland USA; ^7^ British Heart Foundation Cardiovascular Epidemiology Unit, Department of Public Health and Primary Care University of Cambridge Cambridge UK; ^8^ Heart and Lung Research Institute University of Cambridge Cambridge UK; ^9^ Department of Epidemiology, School of Public Health Nanjing Medical University Nanjing China; ^10^ Department of Biostatistics and Center for Statistical Genetics University of Michigan Ann Arbor Michigan USA; ^11^ Programs in Metabolism and Medical and Population Genetics Broad Institute of Harvard and MIT Cambridge Massachusetts USA; ^12^ Diabetes Unit and Center for Genomic Medicine Massachusetts General Hospital Boston Massachusetts USA; ^13^ Centre for Genetics and Genomics Versus Arthritis, Centre for Musculoskeletal Research, Division of Musculoskeletal and Dermatological Sciences University of Manchester Manchester UK; ^14^ TUM School of Medicine and Health Technical University of Munich and Klinikum Rechts der Isar Munich Germany

**Keywords:** genetic associations, genomic control, genomic inflation, GWAS meta‐analysis, LD‐score regression

## Abstract

Inflation in genome‐wide association studies (GWAS) summary statistics represents a major challenge, for which correction methods have been developed. These include the genomic control (GC) method, which uses the λ‐value to correct summary statistics, and the linkage disequilibrium score regression (LDSR) method, which uses the LDSR intercept. By using type 2 diabetes (T2D) as an exemplar, we explore factors influencing λ‐values and the impact of these corrections on association signals. We find that larger sample sizes increase λ‐values due to increased captured polygenicity, while including lower frequency variants decreases λ‐values due to reduced power. Comparing T2D genetic associations described in overlapping GWAS meta‐analyses of increasing sample size, we find that GC correction reduces the false positive rate and leads to the loss of robust associations. In one of the largest meta‐analysis, GC correction results in 39.7% loss of independent loci, substantially reducing the number of detected associations. In comparison, the LDSR intercept correction leads to a loss of up to 25.2% of the independent loci, being therefore less conservative than the GC correction. We conclude that in large, well‐powered GWAS meta‐analysis of polygenic traits, both GC and LDSR intercept correction leads to power loss, highlighting the need for improved genomic inflation correction methods.

## 1 Introduction

The genetic architecture of polygenic diseases is complex, and underpinned by the contribution of multiple variants of varying frequency and effect size (Boyle et al. 2017). Genome‐wide association studies (GWAS) have been successful in identifying associations between genomic loci and complex traits (Sollis et al. 2022). The power of these association studies has been further enhanced by meta‐analyses, which combine association summary statistics from multiple GWAS and therefore increase sample size and statistical power. When conducting a GWAS, a key consideration is to differentiate between true associations and false positives due to an inflation of the test statistics, which could arise from systematic bias. Such bias can be due to a variety of factors that, for example, contribute to population stratification, including differences in allele frequencies and linkage disequilibrium (LD) structure across populations, as well as cryptic relatedness between the participants (Voight and Pritchard 2005; Price et al. 2010). These biases are usually corrected by including principal components capturing population stratification as covariates, and/or by using mixed models to account for population structure and/or relatedness (Price et al. 2010).

The genomic control (GC) correction method has been developed to quantify systematic inflation and correct for it (Devlin and Roeder 1999). It relies on the genomic inflation factor, also called the *λ*‐value, which is defined as the ratio between the median of the observed *χ* ^2^‐association test statistic distribution to its theoretical counterpart under the null hypothesis of no association, where a value greater than 1 is indicative of an inflation in the test statistics (Devlin and Roeder 1999). A key assumption behind this calculation is that only a limited proportion of the variants in the genome are associated with the trait of interest, with most of the genome being under the null model of no association. This assumption has been challenged in recent large genetic studies of complex diseases, where it has been observed that the number of genomic associations detected increases linearly with GWAS sample size, especially due to the detection of variants with smaller effect sizes (Canela‐Xandri et al. 2018). This underlines the polygenicity of complex traits and the contribution of a high number of variants with small individual effects (Visscher et al. 2021). Accordingly, *λ*‐values have increased in studies with larger sample sizes identifying larger proportions of the genome to be associated with the trait (Yang et al. 2011). Intuitively, a larger *λ*‐value will result in a more severe GC correction and may be overconservative (Yang et al. 2011; Wang et al. 2012). Yet, it is still frequent practice to GC correct the summary statistics at the individual study level (single GC correction) as well as to GC correct GWAS meta‐analysis summary statistics (double GC correction) by the respective *λ*‐values. However, it is challenging to discriminate between a high *λ*‐value that is due to confounding or due to high polygenicity of the trait. To tackle this problem, the LD score regression (LDSR) correction method, based on LD information and the distribution of *χ* ^2^‐association test statistics, has been developed and widely adopted to measure inflation and correct summary statistics (Bulik‐Sullivan et al. 2015). This method computes LD scores that capture the likelihood of a variant tagging a true causal variant depending upon the degree of LD with its neighboring variants. LDSR has several key assumptions including that the trait being studied is polygenic, that is, each variant contributes a small effect, the LD scores are estimated from an appropriate reference population and variants with higher LD score should have proportionally larger effect sizes on the trait. A regression of *χ* ^2^‐association test statistics against LD scores is performed, where an intercept value greater than 1 indicates confounding. The LDSR intercept represents the expected test statistics for variants with LD score of zero and can be used to correct the inflation in the *χ* ^2^ test statistics similarly to how the *λ*‐value is used in GC correction. On the other hand, the slope of the regression line represents the heritability of the trait, which is not independent from the intercept. While it has been established that the λ‐value is sensitive to sample size and polygenicity, the potential loss of association signals and independent loci that could result from GC correction in such studies, and whether LDSR intercept correction could represent an effective alternative, remain questions of interest for the scientific community. Here, we investigate the effect of both correction methods using three of the largest GWAS meta‐analyses of T2D (Mahajan et al. 2018; Mahajan et al. 2022; Suzuki et al. 2024). Our approach entails analysis of GWAS meta‐analyses of various sample sizes to ascertain genomic variants consistently associated with T2D across GWAS meta‐analysis and how they are impacted by inflation correction methods.

## 2 Methods

### 2.1 Type 2 Diabetes GWAS Meta‐Analysis Summary Statistics

We used three of the largest T2D GWAS meta‐analyses from the DIAMANTE Consortium and the Type 2 Diabetes Global genomics Initiative (T2DGGI), all BMI‐unadjusted, which we referred to as DIAMANTE‐18, DIAMANTE‐22, and T2DGGI‐24 (Mahajan et al. 2018; Mahajan et al. 2022; Suzuki et al. 2024). We used only the European‐specific analysis from the DIAMANTE‐22 and the T2DGGI‐24 studies, as these comprise the largest subset of the multi‐ancestry dataset and enabled us to compare findings in a similar ancestry group across the three meta‐analyses (the DIAMANTE‐18 study is entirely based on European‐ancestry GWAS). Additional information about the European subset of each study is provided in Supporting Information S1: Table S1. To ensure a fair comparison in our analysis, we did not use the λ‐values reported by the respective study authors as they were based on different filtering strategies across studies, especially in DIAMANTE‐18. Instead, we chose to perform our analysis on the intersecting set of variants across the three studies, corresponding to 10,269,674 variants. The description of the three studies, including the sample size, the number of total and genome‐wide significant variants in our analysis, the method of correction (as reported in the original study), the *λ* value (computed on the shared set of variants or reported in the original study), and the number of independent loci as reported in the original study is provided in Table 1. The DIAMANTE‐18 and DIAMANTE‐22 meta‐analysis summary statistics only report double GC‐corrected *p* values. We uncorrected the *p* values by calculating the *χ*²‐statistics corresponding to the double GC‐corrected *p* values and by multiplying them by the *λ*‐value reported in each study (Table 1). These *χ*²‐statistics were used to determine the uncorrected *p* values. We then calculated the inflation on the shared set of variants using the GC method to determine the new *λ* values (Table 1). Finally, we re‐corrected the *p* values using these new *λ*‐values to obtain the new double GC‐corrected *p* values for our analysis. The impact of genomic inflation correction was not assessed in the T2DGGI‐24 meta‐analysis as it is the latest T2D GWAS meta‐analysis. This study, which report uncorrected *p* values, was employed to ascertain high confidence variants in the DIAMANTE‐18 and the DIAMANTE‐22 meta‐analyses.

**Table 1:** Study characteristics of the three T2D GWAS meta‐analyses included in our study.

Name of study	Variants filtering in original study	Correction for population structure	*λ*‐value (original study)	*λ*‐value (new)	Loci identified (original study)	Significant associations (*p* < = 5 × 10^−8^)
**DIAMANTE‐18** (74,124 cases, 824,006 controls)	HRC reference panel	Double GC correction	1.013	1.387	231	19,328
**DIAMANTE‐22** (80,154 cases, 853,816 controls)	Overlap of 1000 G and HRC, MAF > 0.5% in at least one ancestry group	Double GC correction	1.096	1.402	277	20,925
**T2DGGI‐24** (242,283 cases, 1,569,734 controls)	MAF ≥ 0.5% in at least one of the five ancestry groups from 1000 G	Individual study level correction using LDSR intercept	1.283	1.676	611	64,944

*Note:* Double GC correction in GWAS meta‐analysis means applying GC correction to the individual GWAS as well as to the meta‐analysis summary statistics. *λ*‐value (original study) indicates the *λ*‐value reported by the authors of the original study. *λ*‐value (new) indicates the recalculated *λ*‐value on the selected set of 10,269,674 variants included across the three meta‐analyses. The substantial difference between the original and the new λ‐values is due to the loss of low‐frequency genetic variants in our dataset compared to the original meta‐analysis.

### 2.2 Calculating the *λ*‐Value and the LDSR Intercept

The GC method computes the λ‐value which is defined as follows:

$$\lambda \text{value}=\frac{\mathrm{Median}(\mathrm{observed}{\;\chi }^{2}\text{test statistics})}{\mathrm{Median}(\mathrm{expected}\;\chi^{2}\text{distribution})}$$

In this equation, the median of expected *χ* ^2^‐distribution is 0.454, corresponding to a χ^2^‐distribution with one degree of freedom. For case‐control GWAS, testing for association under an additive model follow a *χ* ^2^‐distribution with one degree of freedom. To assess the effect of LDSR intercept correction, we ran LDSR using the LDSC software (version 1.0.1) with the ‐‐h2 option and calculated the LDSR intercept (Bulik‐Sullivan et al. 2015). Since we analyzed only the European subsets of the meta‐analyses, we employed precomputed LD scores from the 1000 Genomes European ancestry haplotypes (Auton et al. 2015). The LDSR intercept was used to correct the uncorrected *p* values from the DIAMANTE‐18 and the DIAMANTE‐22 meta‐analysis summary statistics, following the same approach as the GC correction. The impact of correction methods was not assessed in the T2DGGI‐24 study. Further, the λ‐value and the LDSR intercept were recomputed after removing variants with a minor allele frequency (MAF) below 0.5%, 1%, 5% and 10% by considering the MAF provided in the respective summary statistics.

### 2.3 Estimating Polygenicity of Type 2 Diabetes

We consider here the polygenicity as a reflection of the proportion of the genome significantly associated with T2D. It is a fixed quantity, which is increasingly captured with larger sample size of GWAS. The variance explained by polygenic factors can be captured by calculating the correlation between the χ^2^‐values and the LD scores of the variants. We computed this quantity using summary statistics from the meta‐analyses from the DIAMANTE‐18, DIAMANTE‐22 and T2DGGI studies within each genetic ancestry group (Mahajan et al. 2018; Mahajan et al. 2022; Suzuki et al. 2024). The effective sample size of the ancestry specific meta‐analyses was calculated using the formula (Willer et al. 2010):

$$N_{\text{effective}}=\frac{4}{\frac{1}{N_{\mathrm{cases}}}+\frac{1}{N_{\mathrm{controls}}}}$$

### 2.4 Confirmation of Associations Across Type 2 Diabetes Meta‐Analyses

We evaluated the impact of the GC and LDSR intercept corrections on the T2D GWAS meta‐analyses. The impact of employing either of the two methods was studied in the smaller meta‐analyses while, the larger meta‐analyses were employed to ascertain high confidence associations. We performed pairwise analyses between T2D meta‐analyses and investigated associations which are confirmed between smaller T2D GWAS meta‐analysis and a larger one (DIAMANTE‐18 was analyzed with respect to DIAMANTE‐22 and T2DGGI‐24, and DIAMANTE‐22 was analyzed with respect to T2DGGI‐24). We refer to these high confidence associations as “robust associations” in the sense that they remain significant with a concordant direction of effect across successive GWAS meta‐analyses with increasing sample size. Note however that the term “robust associations” has a statistical rather than biological meaning as there was no validation in an experimental setting. Robust associations are defined as genetic variants significant at the genome‐wide significance threshold of 5 × 10^−8^ in the earlier and the later study with a consistent direction of effect between the two. The percentage of robust associations between the two studies is computed as follows:

$$\%Robust\;associations=\frac{Total\;number\;of\;robust\;associations\;in\;earlier\;study}{Total\;number\;of\;significant\;associations\;in\;earlier\;study}\times 100$$

For each of the three analyses, we corrected only the earlier study, while the later study is left uncorrected. To assess the impact of correction in the earlier study, the percentage of robust associations is calculated for uncorrected and corrected GWAS meta‐analysis summary statistics of the earlier study.

### 2.5 Investigating Chromosomal Confirmation Rate Using a Leave‐One‐Chromosome‐Out (LOCO) and Random Sampling Approach

To assess whether confirmation rates significantly vary across chromosomes, we employed a LOCO framework. Using the DIAMANTE‐18 and DIAMANTE‐22 meta‐analyses as an example, we first identified the number of genome‐wide significant variants on each chromosome in DIAMANTE‐18 and determined how many were confirmed in DIAMANTE‐22 (methods). Next, we randomly sampled an equal number of genome‐wide significant variants from the rest of the genome, excluding the chromosome of interest, and assessed the corresponding confirmation rate. We then computed an empirical *p* value, denoted as $p_{\mathrm{chr}}$, as following:

$$p_{\mathrm{chr}}=\frac{\sum_{s=1}^{N}\min\left(I\left(C_{s}\leq C_{\mathrm{obs},\mathrm{chr}},C_{s}>C\right)\right)}{N}$$

With *N* being the total number of samplings here corresponding to 100,000, I() the indicator function, $C_{s}$ the confirmation rate for the sampling s, and $C_{\mathrm{obs},\mathrm{chr}}$ the confirmation rate observed for the investigated chromosome. This analysis was conducted for all chromosomes except chromosome 21, which lacked genome‐wide significant variants in the DIAMANTE‐18 and the DIAMANTE‐22 meta‐analyses. We repeated the same for other pairwise analyses considered in our study, i.e., DIAMANTE‐18 with respect to T2DGGI‐24, and DIAMANTE‐22 with respect to T2DGGI‐24. Further, to assess the effect of correction methods on chromosomal confirmation rate, we performed the described analysis on summary statistics after GC or LDSR intercept correction.

### 2.6 False Positive Rates and the True Positive Rates

To assess the relative cost of losing robust associations in comparison to removing false associations, which is the main aim of the two correction methods, we computed the false positive rate and the true positive rate on the aforementioned pairwise analyses. False positive associations correspond to genetic variants genome‐wide significant in the earlier study but not in the later study, or genome‐wide significant in both but with inconsistent direction of effect. True positive associations correspond to variants genome‐wide significant in both studies with consistent direction of effect. False negative associations correspond to variants genome‐wide significant only in the later study and true negative associations to variants not genome‐wide significant in either study. We then calculate the false positive rate and the true positive rate as:

$$\mathrm{False Positive Rate}=\frac{\text{False Positive}}{\text{False Positive}+\text{True Negative}}$$

$$\text{True Positive Rate}\;=\frac{\text{True Positive}}{\text{True Positive}+\text{False Negative}}$$

### 2.7 Assessing the Loss of Independent Loci

In addition to describing the loss of robust associations from the previous analyses, we investigated if this leads to a loss of an independent locus. An independent associated locus is a genomic region comprising multiple variants in high LD, which are significantly associated with the trait of interest after accounting for other nearby genetic variants. It is described using its chromosomal location, genomic length (usually 1 Mb), and index variant (usually the most significant variant). To do so, we used the study‐defined index variants as proxy for the DIAMANTE‐18 and the DIAMANTE‐22 studies. To assess whether the lost robust associations lead to a loss of independent loci, we used the following analysis workflow (Supporting Information S1: Figure S1) for each chromosome:

1. We ran LD clumping using PLINK for DIAMANTE‐18 and DIAMANTE‐22 meta‐analyses using 1000 genomes reference data for Europeans (Purcell et al. 2007).
2. We calculated the genomic boundaries of the LD clumps as the minimum and maximum genomic position in the clump.
3. We assessed the study defined T2D index variants to determine the specific LD clump they belong to.
4. Next, we determined if the lost robust associations fall within the same LD clumps as the study defined T2D index variants.
5. Lost robust associations part of LD clumps different from those of the study‐defined index variants potentially represent lost independent loci.
6. Finally, we determined the number of unique LD clumps for these lost robust associations untagged by study defined T2D index variants.

In this, meta‐analysis variants not part of the LD reference panel and falling outside the LD clumps were defined as single variant clumps. These variants were excluded when assessing the loss of independent loci. The LD clumping in PLINK was ran in windows of 500 kb with an r2 threshold of 0.20 and significance thresholds of 5 × 10^−8^ and 5 × 10^−6^ for the index and secondary signals, respectively.

Further, lost robust associations located at the major histocompatibility complex region chr6:28,477,797‐33,448,354 (GRCh37) were removed because the region is characterized by extremely high LD and high density of genes which makes it difficult to distinguish independent association signals (de Bakker and Raychaudhuri 2012).

To calculate the percentage of lost independent loci, we divided the number of independent loci lost due to correction by the total number of significant independent loci before correction. The total number of independent loci before correction was determined by summing the lost independent loci and the independent loci identified in the smaller study for the respective analysis.

## 3 Results

### 3.1 Factors Contributing to Increased Genomic Inflation

**Figure 1:**
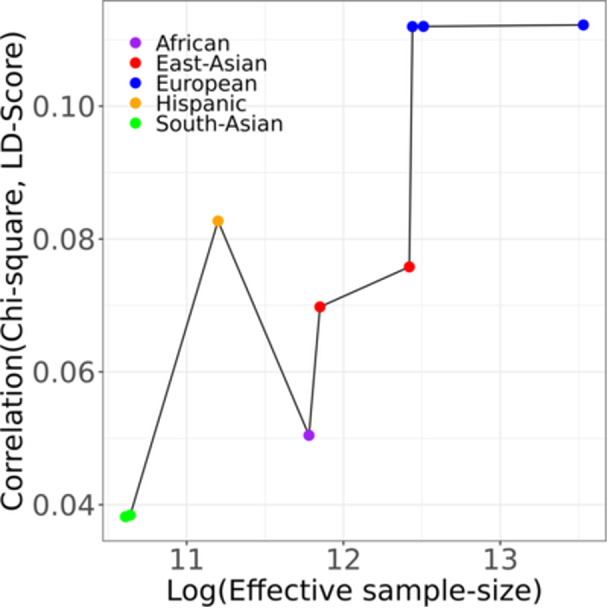
Effective sample size against the correlation between χ^2^‐values and LD scores from ancestry‐specific meta‐analyses part of DIAMANTE‐18, DIAMANTE‐22 and T2DGGI‐24 meta‐analyses.

We started by investigating factors driving the lambda values by analyzing the 10,269,674 variants included across the three meta‐analyses. We observe a linear trend between the effective sample size of the meta‐analysis and the *λ*‐values (Supporting Information S1: Figure S2), as previously reported (Yang et al. 2011). We next investigated the impact of allele frequency cutoffs by recomputing *λ*‐values using varying MAF thresholds (methods). We observed that *λ*‐values increase as rare and low‐frequency variants are removed (Supporting Information S1: Figure S3A), likely reflecting lower statistical power (Pritchard 2001; Li and Leal 2008). This was corroborated by the fact that the *λ*‐value stabilizes after removing variants with a MAF lower than 5% for each of the three meta‐analyses (Supporting Information S1: Figure S3). The same was found to be true for LDSR intercept, although this increase was lower compared to the *λ*‐value (Supporting Information S1: Figure S3B). Finally, we investigated the impact of the polygenicity of the trait. Polygenicity is a fixed quantity associated with complex traits, for which we have used as a proxy the proportion of variance in the χ^2^‐statistics explained by polygenetic factors (methods). We find a general positive trend between this quantity and the effective sample size of the respective meta‐analyses (Figure 1). This highlights that polygenicity is revealed with increasing sample size and contributes to the rise in genomic inflation, concordant with results from Yang et al. (Yang et al. 2011).

### 3.2 Correction Post Meta‐Analysis Leads to Loss of Robust Associations

**Figure 2:**
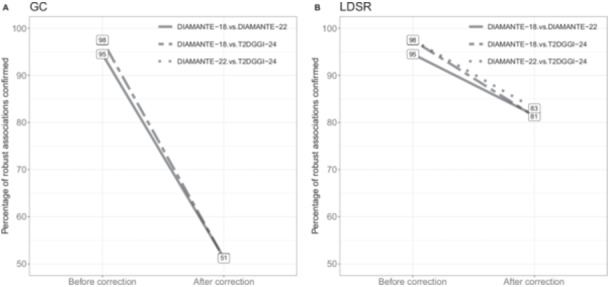
The proportion of robust associations before and after correcting the earlier meta‐analysis in the three analyses. (A) Correction using the GC method. (B) Correction using the LDSR method.

While previous studies have investigated factors leading to an increase in genomic inflation, no study has assessed the impact of the corresponding correction on the potential loss of genetic signals. To investigate this question, we assessed whether GC‐correcting the *p* values would lead to a loss of robust associations. For this, we conducted pairwise analyses between two successive studies (methods). We calculated the proportion of robust associations in the earlier study confirmed in the later study both before and after applying the correction method. As shown in Figure 2A, we observed a substantial decline in the proportion of robust associations after GC correction, with as much as 49.08% of the robust associations being lost when analyzing DIAMANTE‐22 and T2DGGI‐24 (Table 2). The robust associations that were lost show *p* values close to the genome‐wide significance threshold, explaining their loss after correction (Supporting Information S1: Figure S4). Moreover, examining the DIAMANTE‐18 vs DIAMANTE‐22 analysis, we found that out of the 8359 robust associations lost due to GC correction, 8,015 were retrieved in the larger T2DGGI‐24 meta‐analysis, where they reached genome‐wide significance. This demonstrates the power of sample size in GWAS and confirms that the lost robust associations indeed represent high confidence associations. We compared these findings with a correction based on the LDSR, which integrates LD information to better estimate inflation compared to the λ‐value. For the DIAMANTE‐18 and the DIAMANTE‐22 meta‐analyses, we report lower values of the LDSR intercept compared to the *λ*‐values (Table 2). By applying a similar procedure as for the GC correction, we again observed a decrease of the proportion of robust associations following correction (Table 2, Figure 2B), but which was less severe than when using the GC correction for all analyses. We observed that robust associations that did not remain significant after correction had *p* values near the genome‐wide significance threshold (Supporting Information S1: Figure S5). Of the 2509 robust associations lost following LDSR intercept correction between the DIAMANTE‐18 and the DIAMANTE‐22 meta‐analysis, 2367 were recovered in the larger T2DGGI‐24 meta‐analysis. Altogether, we show that both correction methods lead to a loss of robust associations, although this loss is notably smaller with the LDSR intercept correction.

**Table 2:** Loss of robust associations after GC and LDSR intercept correction in the three analyses.

Analyzed studies	Earlier study		Robust associations before correction	Robust associations after correction		Robust associations lost	
	*λ*‐value	LDSR		GC	LDSR	GC	LDSR
**DIAMANTE‐18 versus DIAMANTE‐22**	1.387	1.097	18,266	9907	15,757	8359	2509
**DIAMANTE‐18 versus T2DGGI‐24**	1.387	1.097	18,830	9893	15,713	8937	3117
**DIAMANTE‐22 versus T2DGGI‐24**	1.402	1.091	20,405	10,390	17,364	10,015	3041

*Note:* LDSR indicates LDSR intercept calculated on the smaller study in the pairwise analysis.

### 3.3 Robust Associations Lost Only to GC Correction Have Significantly Lower *p* values

**Figure 3:**
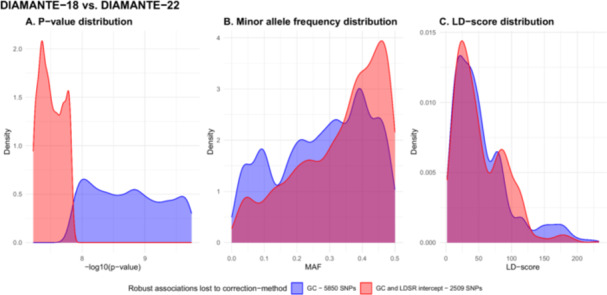
Distributions of *p* values, MAF and LD‐scores for robust associations lost only to GC correction compared to those lost to both GC and LDSR intercept correction in the DIAMANTE‐18 and the DIAMANTE‐22 analysis. *p* value distribution: *t*‐test *p* < 2 × 10^−16^. Minor allele frequency distribution: *t*‐test *p* < 2 × 10^−16^. LD score distribution: *t*‐test p = 0.346. (A) *P*‐value distribution (B) Minor allele frequency distribution (C) LD‐score distribution 0.015

Since the λ‐value is substantially higher than LDSR intercept value for the DIAMANTE‐18 and the DIAMANTE‐22 meta‐analyses (Table 2), robust associations lost to LDSR intercept correction are a subset of those lost to GC correction in the three analyses. Given this observation, we analyzed associations lost only to GC correction compared to those lost to both correction methods. When analyzing DIAMANTE‐18 and DIAMANTE‐22, we found 5850 robust associations that were lost only to GC correction and 2509 robust associations that were lost to GC correction and LDSR intercept correction. As expected, we found that robust associations lost only to GC correction have significantly lower *p* values compared to robust associations lost to GC and LDSR intercept correction (*t*‐test *p* < 2 × 10^−16^, Figure 3A, Supporting Information S1: Figures S6 and S7). On average, we observed lower MAF for the associations lost only due to GC correction compared to associations lost with both correction methods (*t*‐test *p* < 2 × 10^−16^, Figure 3B). We did not find significant differences in LD scores between associations lost only due to GC correction or to both correction methods (*t*‐test *p* = 0.346, Figure 3C). Similar results were observed in the DIAMANTE‐18 vs. T2DGGI‐24 analysis (Supporting Information S1: Figure S6), while in the DIAMANTE‐22 vs. T2DGGI‐24 analysis, we observed significant differences in both the *p* value and LD score distributions (Supporting Information S1: Figure S7).

### 3.4 Confirmation Rates Vary Across the Genome

We observed that confirmation rates vary across the genome, with or without correcting the *p* values for genomic inflation (Table 3 and Supporting Information S1: Table S2). To assess whether specific chromosomes show confirmation rates significantly different from the rest of the genome, we employed a LOCO approach with 100,000 samplings (methods). Across all three uncorrected analyses, we consistently observed that the confirmation rates for chromosomes 1 and 14 did not differ from the rest of the genome (Supporting Information S1: Table S2). In contrast, chromosomes 10, 12, 13, 15, 16 and 17 exhibited significantly higher confirmation rates, while chromosome 13 had a significantly lower confirmation rate compared to the rest of the genome. We then examined whether these confirmation patterns persisted after applying GC or LDSR intercept correction. Following GC correction, chromosomes 10, 12, 15, 16 and 17 continue showing a higher confirmation rate, while chromosome 13 showed lower confirmation rate compared to the rest of the genome (Table 3). After LDSR intercept correction, only chromosomes 10, 12, and 15 continue showing significantly higher confirmation rates than the rest of the genome. While no chromosome showed a lower confirmation rate than the rest of the genome (Table 3).

**Table 3:** Chromosomal confirmation rate assessed using a LOCO framework with 100,000 genome‐wide random samplings (after GC or LDSR intercept correction) in the DIAMANTE‐22 vs T2DGGI‐24 analysis as an example.

Chr	Observed confirmation rate		Mean confirmation rate in the rest of the genome from simulation (*p* value)	
	DIAMANTE‐22 versus T2DGGI‐24		DIAMANTE‐22 versus T2DGGI‐24	
	GC correction	LDSR intercept correction	GC correction	LDSR intercept correction
1	0.81	0.88	0.80 (0.36)	0.88 (0.10)
2	0.73	0.86	0.71 (**< 1e‐6**)	0.85 (**< 1e‐6**)
3	0.77	0.83	0.75 (**1e‐5**)	0.82 (**< 1e‐6**)
4	0.68	0.7	0.74 (**< 1e‐6**)	0.73 (**< 1e‐6**)
5	0.73	0.83	0.77 (**< 1e‐6**)	0.85 (**< 1e‐6**)
6	0.73	0.88	0.75 (**< 1e‐6**)	0.90 (**< 1e‐6**)
7	0.79	0.91	0.79 (0.24)	0.90 (2e‐2)
8	0.78	0.75	0.77 (1e‐2)	0.74 (**1e‐3**)
9	0.76	0.74	0.75 (4e‐2)	0.74 (0.11)
10	0.75	0.91	0.73 (**< 1e‐6**)	0.90 (**7e‐4**)
11	0.74	0.72	0.73 (**< 1e‐6**)	0.71 (**2e‐4**)
12	0.77	0.83	0.75 (**< 1e‐6**)	0.81 (**1e‐5**)
13	0.78	0.69	0.86 (**< 1e‐6**)	0.69 (0.28)
14	0.8	0.6	0.80 (0.49)	0.60 (9e‐2)
15	0.82	0.83	0.80 (**< 1e‐6**)	0.81 (**2e‐5**)
16	0.72	0.86	0.70 (**1e‐4**)	0.85 (**2e‐3**)
17	0.76	0.83	0.74 (**1e‐5**)	0.82 (2e‐3)
18	0.77	0.73	0.75 (7e‐3)	0.72 (2e‐2)
19	0.75	0.82	0.73 (**5e‐4**)	0.80 (1e‐2)
20	0.72	0.79	0.75 (**8e‐4**)	0.79 (0.20)
22	0.74	0.75	0.76 (**5e‐4**)	0.75 (0.20)

*Note:* Empirical *p* values highlighted in bold are significant at a Bonferroni significance threshold of 0.0023 corrected for the 21 chromosomes.

Abbreviation: Chr, chromosome.

### 3.5 Correction Marginally Reduces the False Positive Rate and Decreases the True Positive Rate

The main purpose of applying correction methods like GC and LDSR is to eliminate false positive associations while retaining robust associations. We therefore evaluated whether the loss of robust associations was compensated by a strong decrease in false positive signals by computing the false positive rate and the true positive rate across the pairwise analyses. While applying the GC correction led to a complete elimination of false positive associations in the DIAMANTE‐18 vs the DIAMANTE‐22 meta‐analysis (Table 4), the true positive rate fell drastically in the same analysis. When using LDSR intercept correction, the false positive rate was 10 times lower than before correction, and the decrease in the true positive rate was smaller compared to the GC correction (Table 4). A similar pattern was observed in the DIAMANTE‐18 vs the T2DGGI‐24 and the DIAMANTE‐22 vs the T2DGGI‐24 analyses (Table 4). Despite the observed decrease in the false positive rate, we show that this rate was small even before applying any correction in the different meta‐analyses. This observation is likely due to the individual studies contributing to the meta‐analysis being corrected at the individual study level using GC. In summary, we conclude that applying the GC correction method in large meta‐analyses reduces the false positive rate, although being already low, and substantially decreases the true positive rate, resulting in a loss of robust associations. In concordance with the previous result of our study, the decrease in true positive rate was smaller using the LDSR intercept correction than the GC correction.

**Table 4:** False positive rate and true positive rate before and after GC and LDSR intercept correction in the three analyses.

	GC correction				LDSR intercept correction			
	False positive rate		True positive rate		False positive rate		True positive rate	
Before/After correction	**Before**	**After**	**Before**	**After**	**Before**	**After**	**Before**	**After**
**DIAMANTE‐18 versus DIAMANTE‐22**	0.0001	0	0.87	0.47	0.0001	0.00001	0.87	0.75
**DIAMANTE‐18 versus T2DGGI‐24**	0.00004	0.000001	0.28	0.15	0.00004	0.00002	0.28	0.24
**DIAMANTE‐22 versus T2DGGI‐24**	0.00005	0.000004	0.31	0.15	0.00005	0.00002	0.31	0.26

### 3.6 Correction Post Meta‐Analysis Leads to Loss of Independent Loci

As tested variants in GWAS meta‐analyses are correlated due to LD, we investigated whether the lost robust associations lead to a loss of independent loci, and thus biological insight into the disease. To do so, we evaluated if robust associations lost from each of the three analyses were tagged by any of the predefined T2D index variant in the original study (methods). Robust associations that were untagged were further analyzed and a subset of them were found to represent lost independent loci (methods). Comparing the DIAMANTE‐18 and DIAMANTE‐22 studies while excluding single variant clumps, we found that GC and LDSR intercept corrections resulted in the loss of 149 (39.2%) and 90 (23.6%) independent loci from the DIAMANTE‐18 meta‐analysis, respectively (Table 5). When analyzing the DIAMANTE and the T2DGGI‐24 studies, both correction methods showed a loss of large number of loci (Table 5). The DIAMANTE‐22 meta‐analysis showed a loss of 170 loci due to GC correction which corresponds to a loss of 38.0% of the independent loci before correction in the DIAMANTE‐22 study. We observed that both correction methods result in a substantial loss of biological information, but, in concordance with the previous results, the loss was smaller using the LDSR intercept correction compared to GC correction.

**Table 5:** Loss of independent loci after GC and LDSR intercept correction in the three analyses.

Analyzed studies		GC correction			LDSR intercept correction		
	Significant independent loci before correction	Lost robust associations untagged	Independent loci lost	% of lost independent loci	Lost robust associations untagged	Independent loci lost	% of lost independent loci
**DIAMANTE‐18 versus DIAMANTE‐22**	380	1609	149	39.20%	490	90	23.60%
**DIAMANTE‐18 versus T2DGGI‐24**	380	1799	151	39.70%	656	96	25.20%
**DIAMANTE‐22 versus T2DGGI‐24**	447	2593	170	38.00%	1068	102	22.80%

## 4 Discussion

Correction of genomic inflation arising from systematic bias in GWAS is important in genetic association studies. Using T2D as an exemplar polygenic disease and three of the largest T2D GWAS meta‐analyses to date, we explore two widely used correction methods, the GC based on the λ‐value, and the LDSR. We confirm previous observations that λ‐values from meta‐analyses increase with sample size and captured polygenicity of the disease. By further investigating the consequences of correction on associations, we show that it leads to a loss of power, through the loss of confirmed, high confidence associations, as well as independent loci. We further show that, GC correction leads to a larger loss in power compared to LDSR intercept correction, characterized by a smaller loss of robust associations and independent loci.

The emergence of global consortia and the resulting increase in the sample size of large GWAS meta‐analyses have greatly contributed to our understanding of the genetic architecture of complex traits and diseases like height, T2D, and schizophrenia (Trubetskoy et al. 2022; Yengo et al. 2022; Suzuki et al. 2024). This has been accompanied by higher *λ*‐values for traits with a substantial polygenic background. The polygenicity of a trait is a fixed quantity which is being increasingly captured with larger sample size and higher statistical power to detect variants, asymptotically approaching that fixed quantity. In our study, we proxied polygenicity by the correlation between the χ^2^‐values and LD scores from ancestry specific meta‐analyses from DIAMANTE‐18, DIAMANTE‐22, and T2DGGI‐24 studies. Other methods, such as the attenuation ratio, have been developed to estimate polygenic effects (Loh et al. 2018). This method relies on the observed *χ* ^2^‐test statistics, which were not available to us for all the individual T2D GWAS comprising the T2DGGI‐24 meta‐analysis. Related to this limitation, we were unable to compute the correlation between LD score and *χ*² statistics for individual T2D GWAS and relied instead on ancestry‐specific meta‐analysis summary statistics. Our results are in line with the findings of Yang et al. which describes the effect of sample size and polygenicity on *λ*‐value (Yang et al. 2011). Additionally, we show that *λ*‐value and LDSR intercept are sensitive to the variant frequency thresholds used in meta‐analysis, with the inclusion of rarer variants leading to a decreased inflation, with an effect less pronounced on the LDSR intercept. Larger sample size leads to increased statistical power, which is, however, still limited for rare and low frequency variants (Lee et al. 2014; Bomba et al. 2017). Even if detected as genome‐wide significant, such rare variants likely have *p* values close to the significance threshold and will, therefore, also be impacted by GC correction, as highlighted in a previous study by Georgiopoulos & Evangelou (Georgiopoulos and Evangelou 2016).

In addition, we investigate, for the first time, the consequences on information lost from correction. Firstly, we demonstrate that GC correction leads to a loss of robust associations, i.e., associations that were confirmed in a larger GWAS meta‐analysis. By assessing these lost associations in a subsequent study, we further confirm that they correspond to high confidence signals being recapitulated in meta‐analyses with larger sample sizes. Some associations were lost only with GC correction and not LDSR intercept correction due to the larger λ‐values, which were found to show significantly lower *p* values and MAF. Variants with higher MAF are expected to have longer range LD and higher LD scores compared to variants with lower MAF (Gazal et al. 2017; Lee et al. 2018). However, we found no significant difference between the LD scores of associations being lost only to GC correction compared to associations lost to both correction methods. Analyzing the confirmation rate by chromosome reveals distinct patterns, some of which are consistent while others disappear after GC or LDSR intercept correction. Moreover, reflecting these results, we observe that GC correction led to a steep decline in the true positive rate, as well as a reduction in an already low false positive rate. Compared to GC correction, LDSR intercept correction resulted in a lower loss of robust associations and independent loci but still led to a substantial reduction in statistical power, affecting as much as 25.2% of the T2D loci identified in the DIAMANTE‐18 meta‐analysis. Thus, we demonstrate that using either correction method ultimately leads to a substantial loss of potential biological insights.

Although our study demonstrates the effect of GC and LDSR intercept correction in large meta‐analyses, we focus our main comparative analysis only on the European subset of the meta‐analyses to limit the impact of ancestry‐related differences. Further research is needed to assess the impact of correction in other ancestries. However, current sample sizes for these populations are still smaller compared to those in European GWAS. When sample sizes are equalized, we expect the impact to be at least as significant as observed in European ancestry GWAS. This is particularly true for LDSR intercept correction, which relies on LD estimates that are less precise due to underrepresentation of non‐EUR populations in LD reference panels (Appadurai et al. 2023). Leveraging the power of genetic diversity, multi‐ancestry meta‐analysis tools like MR‐MEGA have been key to the discovery of novel genetic loci associated with complex traits (Mägi et al. 2017). The impact of correction in multi‐ancestry meta‐analysis remains to be explored, especially LDSR intercept correction, which is affected by the aforementioned challenges. In the multi‐ancestry meta‐analyses of DIAMANTE‐22 and T2DGGI‐24, the authors reported lower λ‐values when accounting for ancestry‐correlated allelic effect heterogeneity compared to fixed or random effects meta‐analysis. This highlights the need to use specific methods when performing trans‐ancestry meta‐analyses to better model differences across ancestries. Studying variants with heterogenous effect sizes across ancestral groups, which are detectable in larger and more diverse meta‐analyses, is important for understanding the genetic architecture of the disease. Considering this, it would be important to assess the impact of correction on the confirmation of variants with fixed and heterogeneous effects across ancestral groups. Another limitation of our study is the presence of sample overlap among the three meta‐analyses. Specifically, the DIAMANTE‐18 and DIAMANTE‐22 datasets are fully included within the T2DGGI‐24 meta‐analysis. Such overlap can artificially inflate the observed concordance between studies. However, T2DGGI‐24 includes more than three times the number of T2D cases compared to either DIAMANTE studies, resulting in substantially greater statistical power to detect associations (Skol et al. 2006). This large sample size enables the T2DGGI‐24 meta‐analysis to more comprehensively capture the polygenic architecture of T2D, leading to identification of several hundred novel loci—substantially more than either of the DIAMANTE studies (Rotondi and Bull 2013).

The GC method was originally developed under the assumption that more than 50% of the tested genetic variants are not associated with the trait. However, this assumption is increasingly challenged in large‐scale GWAS meta‐analyses of complex traits, where a substantial portion of the genome shows association. As highlighted from our study, further methodological developments to better assess genomic inflation are needed. One potential direction would be to use different percentiles of the test statistic distribution in the GC correction rather than the median. Alternatively, as statistical power depends on the MAF of the variants (Supporting Information S1: Figure S3A), GC corrections could be derived representing different frequency strata. Ideally, new methods should be developed at a finer scale to improve calibration and better capture the genetic architecture of complex traits. As meta‐analyses increase in sample size, they gain greater power to detect associations involving low‐frequency variants. However, many of these variants are absent or poorly represented in reference panels, leading to inaccuracies in LD score estimation. This mismatch can result in inflated estimates of confounding in the LDSR, as the model may misattribute the signal from low‐frequency polygenic effects to confounding bias.

In conclusion, our findings suggest that GC and LDSR intercept correction are not optimal for addressing genomic inflation in large meta‐analyses of polygenic traits, as polygenicity is better accounted for in large meta‐analyses. We demonstrate that although LDSR intercept correction reduces the loss of robust associations and independent loci compared to GC correction, it still leads to a loss of power. We expect these issues to persist in upcoming meta‐analyses, which will be larger and thus capturing more polygenic effects of complex traits, as well as more diverse, making the use of LD‐based methods challenging. We therefore highlight the need for methodological development to better capture and correct for genomic inflation in large GWAS meta‐analyses, including the need of more representative LD reference panels to improve the accuracy of LD score calculations.

## Supporting information


**Supplementary:** Tables and Figures.

## Data Availability

The Jupyter notebooks associated with the analysis are available in: https://github.com/hmgu-itg/lambda_project/.
