# Supplementary material for: Correcting for Genomic Inflation Leads to Loss of Power in Large‐Scale Genome‐Wide Association Study Meta‐Analysis
Source: Genet Epidemiol. 2025 Aug 6;49(6):e70016. doi: 10.1002/gepi.70016 (PMC12327166; doi:10.1002/gepi.70016)
Supplement: Supplementary file 1 — Supplementary: Tables and Figures. [file GEPI-49-0-s001.docx]

# Supplementary Tables and Figures

Supplementary table 1: Additional information on the European subset of the three T2D meta-analyses part of our investigation.

| **T2D meta-analysis** | **Number of individual GWAS** | **Imputation** | | | **Correction of population structure** |
| --- | --- | --- | --- | --- | --- |
|  |  | **Reference panel** | **Software** | **Quality Control** |  |
| DIAMANTE-18 | 32 | HRC, Icelandic panel | Minimac3 or Impute4 | r^2^>0.3 or proper info>0.4 | PC adjustment or mixed model with GRM |
| DIAMANTE-22 | 32 | 1000G, HRC, Icelandic panel, Estonian/ Finnish panel | Minimac3 or Impute4 or Beagle4.1 | r^2^≥0.3 or info≥0.4 | PC adjustment or mixed model with GRM or related individuals excluded |
| T2DGGI-24 | 36 | 1000G, TOPMed, Icelandic panel, HRC, Estonian/ Finnish panel | Minimac3 or Impute4 or Beagle4.1 or Beagle5.1 | r^2^≥0.3 or info≥0.4 | PC adjustment or mixed model with GRM or related individuals excluded |

*All individual GWAS entering the DIAMANTE-18 meta-analysis are analysed in DIAMANTE-22 meta-analysis. All individual GWAS entering the DIAMANTE-22 meta-analysis are analysed in T2DGGI-24 meta-analysis. GRM: genetic relationship matrix; HRC: Haplotype Reference Consortium; PC: Principal Component*

Supplementary table 2: Chromosomal confirmation rate assessed using a LOCO framework with 100K genome-wide random samplings (before correction).

| **Chr** | **Observed confirmation rate** | | | **Mean confirmation rate in the rest of the genome from simulation (P-value)** | | |
| --- | --- | --- | --- | --- | --- | --- |
|  | **DIAMANTE-18**  **vs.**  **DIAMANTE-22** | **DIAMANTE-18**  **vs.**  **T2DGGI-24** | **DIAMANTE-22**  **vs.**  **T2DGGI-24** | **DIAMANTE-18**  **vs.**  **DIAMANTE-22** | **DIAMANTE-18**  **vs.**  **T2DGGI-24** | **DIAMANTE-22**  **vs.**  **T2DGGI-24** |
| 1 | 0.96 | 0.96 | 0.97 | 0.944 (2e-3) | 0.974 (1.9e-2) | 0.975 (0.124) |
| 2 | 0.92 | 0.99 | 1.0 | 0.947 (**<1e-6**) | 0.971 (**<1e-6**) | 0.972 (**<1e-6**) |
| 3 | 0.89 | 0.99 | 0.99 | 0.948 (**<1e-6**) | 0.973 (**<1e-6**) | 0.973 (**<1e-6**) |
| 4 | 0.96 | 0.89 | 0.91 | 0.943 (**1e-3**) | 0.978 (**<1e-6**) | 0.978 (**<1e-6**) |
| 5 | 0.93 | 0.93 | 0.93 | 0.945 (4e-2) | 0.977 (**<1e-6**) | 0.978 (**<1e-6**) |
| 6 | 0.98 | 0.95 | 0.95 | 0.936 (**<1e-6**) | 0.977 (**0.0**) | 0.979 (**<1e-6**) |
| 7 | 0.98 | 0.99 | 0.98 | 0.943 (**<1e-6**) | 0.973 (**8e-5**) | 0.974 (6e-2) |
| 8 | 0.89 | 0.98 | 0.98 | 0.949 (**<1e-6**) | 0.973 (**3e-5**) | 0.974 (4e-3) |
| 9 | 0.99 | 0.99 | 0.99 | 0.943 (**<1e-6**) | 0.973 (**1e-2**) | 0.974 (6e-3) |
| 10 | 0.99 | 0.99 | 0.99 | 0.941 (**<1e-6**) | 0.972 (**<1e-6**) | 0.973 (**<1e-6**) |
| 11 | 0.90 | 0.99 | 0.99 | 0.947 (**<1e-6**) | 0.973 (**<1e-6**) | 0.974 (**<1e-6**) |
| 12 | 0.99 | 1.0 | 0.99 | 0.942 (**<1e-6**) | 0.972 (**<1e-6**) | 0.973 (**<1e-6**) |
| 13 | 0.63 | 0.82 | 0.90 | 0.949 (**<1e-6**) | 0.976 (**<1e-6**) | 0.975 (**<1e-6**) |
| 14 | 0.91 | 0.98 | 0.96 | 0.945 (2.4e-2) | 0.974 (0.41) | 0.975 (0.147) |
| 15 | 0.98 | 0.99 | 0.99 | 0.942 (**<1e-6**) | 0.973 (**0.0**) | 0.973 (**<1e-6**) |
| 16 | 0.97 | 0.99 | 0.99 | 0.944 (**3e-4**) | 0.973 (**8.5e-4**) | 0.974 (**4e-5**) |
| 17 | 0.97 | 0.99 | 0.99 | 0.943 (**<1e-6**) | 0.973 (**1e-5**) | 0.974 (**<1e-6**) |
| 18 | 0.97 | 1.0 | 0.99 | 0.944 (9e-3) | 0.973 (**4.7e-4**) | 0.974 (**1e-3**) |
| 19 | 0.96 | 1.0 | 1.0 | 0.944 (2.1e-2) | 0.973 (**1e-4**) | 0.974 (**4e-5**) |
| 20 | 0.94 | 0.98 | 0.94 | 0.944 (0.42) | 0.974 (0.17) | 0.975 (**6e-4**) |
| 22 | 0.62 | 0.95 | 0.94 | 0.951 (**<1e-6**) | 0.974 (5e-3) | 0.975 (2e-3) |

*Empirical p-values highlighted in bold are significant at a Bonferroni significance threshold of 0.0023 corrected for the 21 chromosomes. Chr: Chromosome*

Supplementary figure 1. Flowchart describing how lost independent loci were defined.


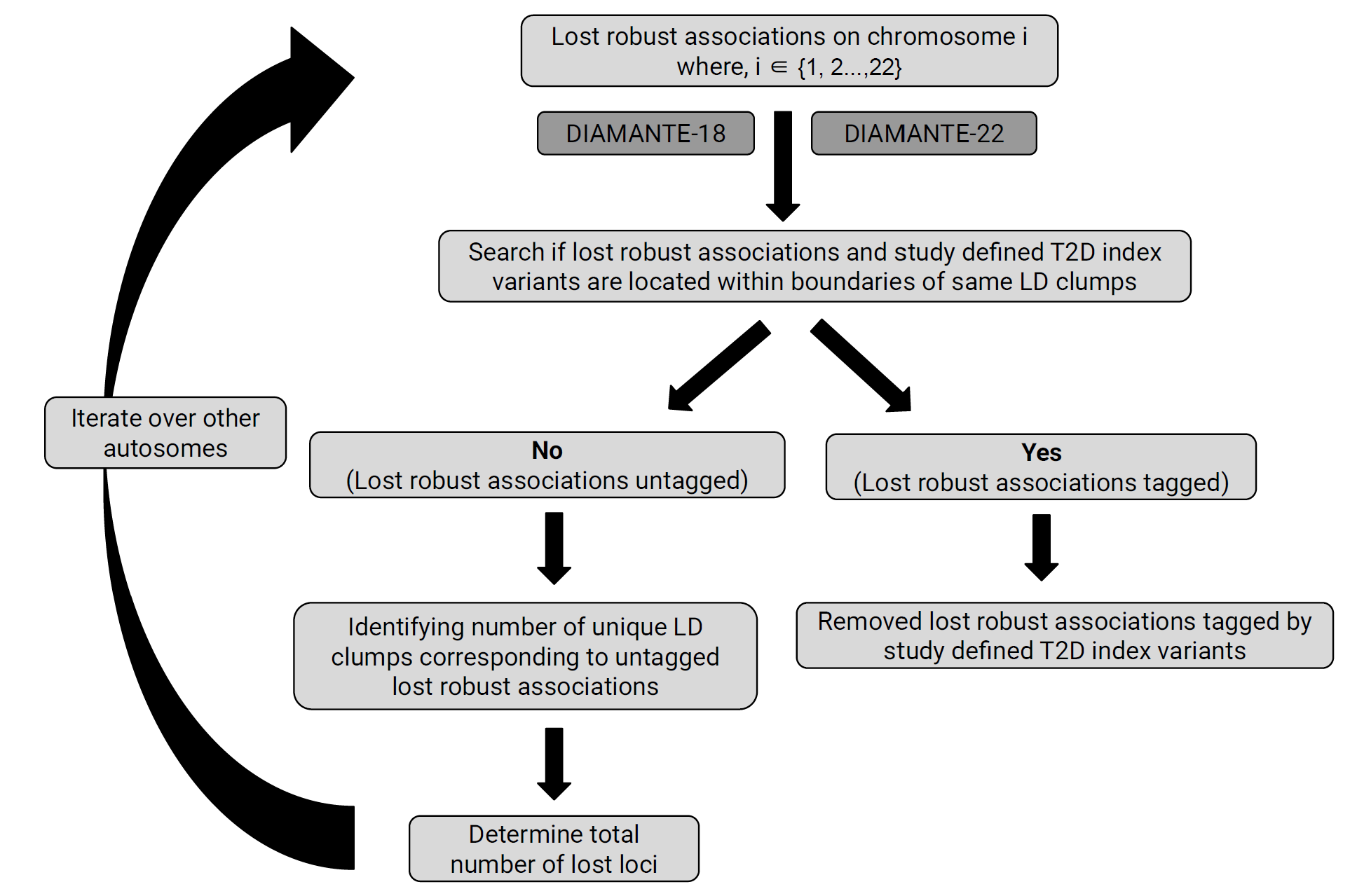


Supplementary Figure 2. λ-values according to the sample size of the three T2D meta-analyses.


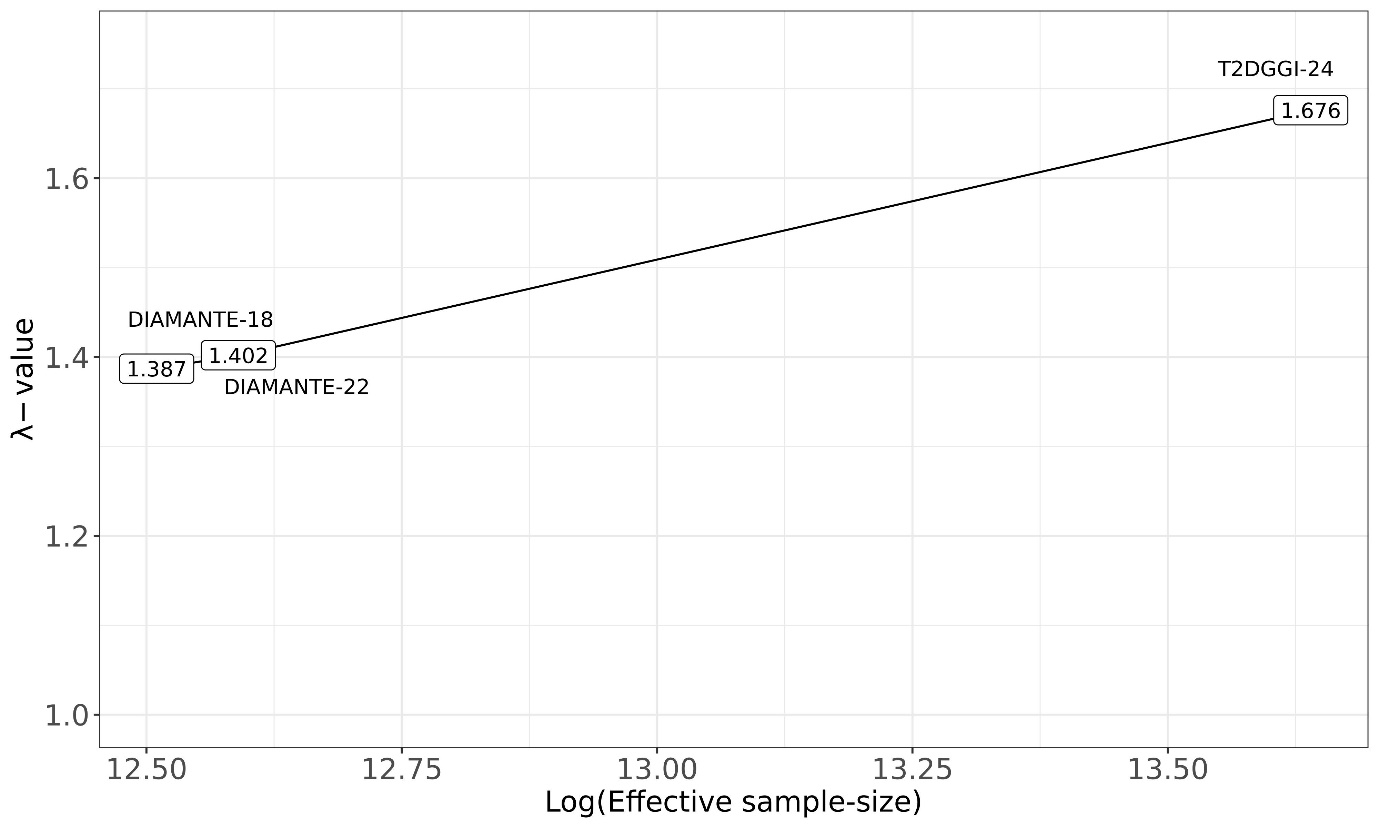


Supplementary figure 3: **A**. λ-values according to the minor allele frequency threshold in the three meta-analyses. **B.** LDSR intercept values according to the minor allele frequency threshold in the three meta-analyses.
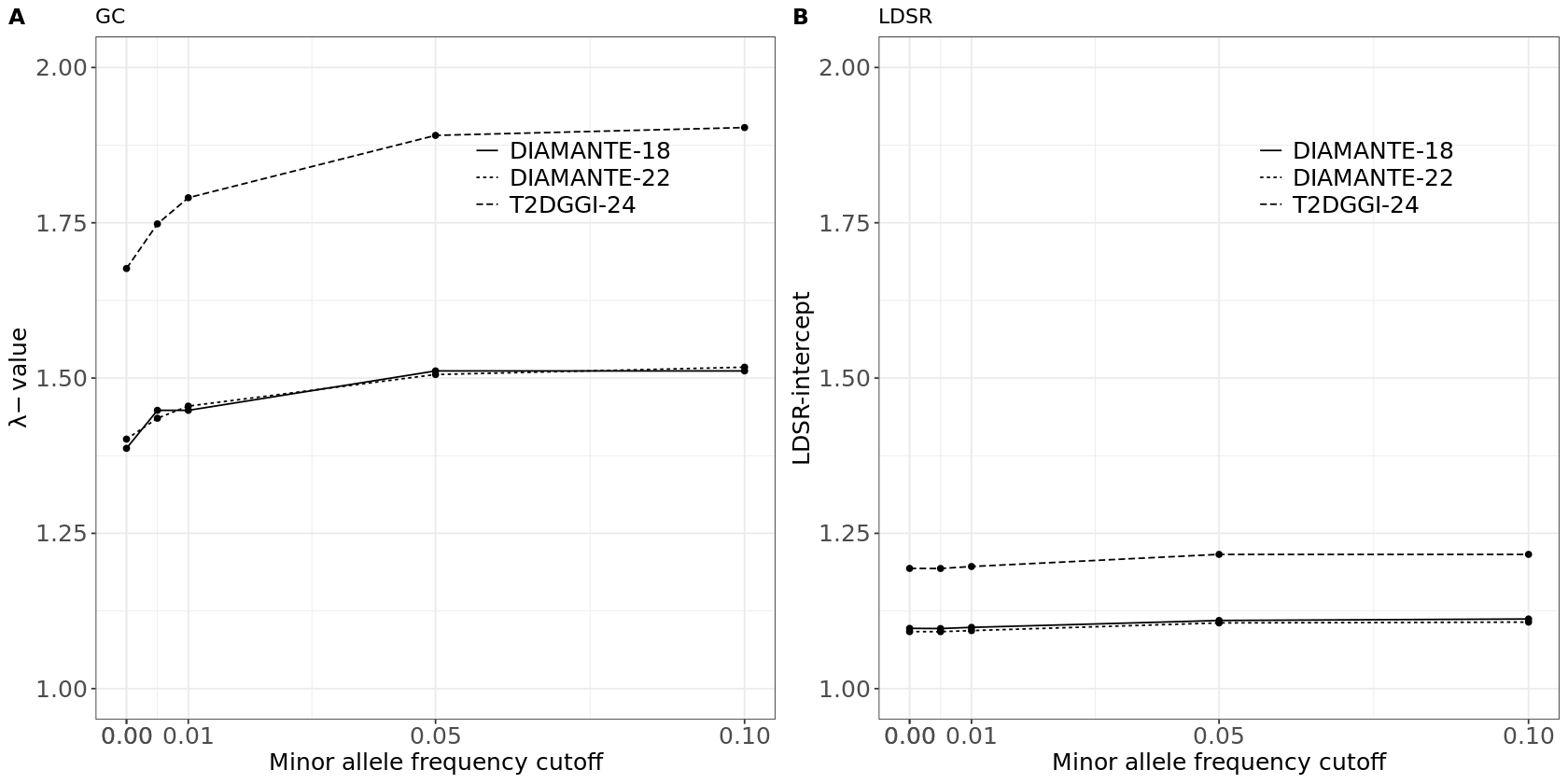


Supplementary figure 4: Correlation plot showing p-values of SNPs across the genome before and after GC correction in the DIAMANTE studies. **A**: DIAMANTE-18 and **B**: DIAMANTE-18: Zoomed-in around the genome-wide significance cutoff. **C**: DIAMANTE-22 and **D**: DIAMANTE-22: Zoomed-in around the genome-wide significance cutoff


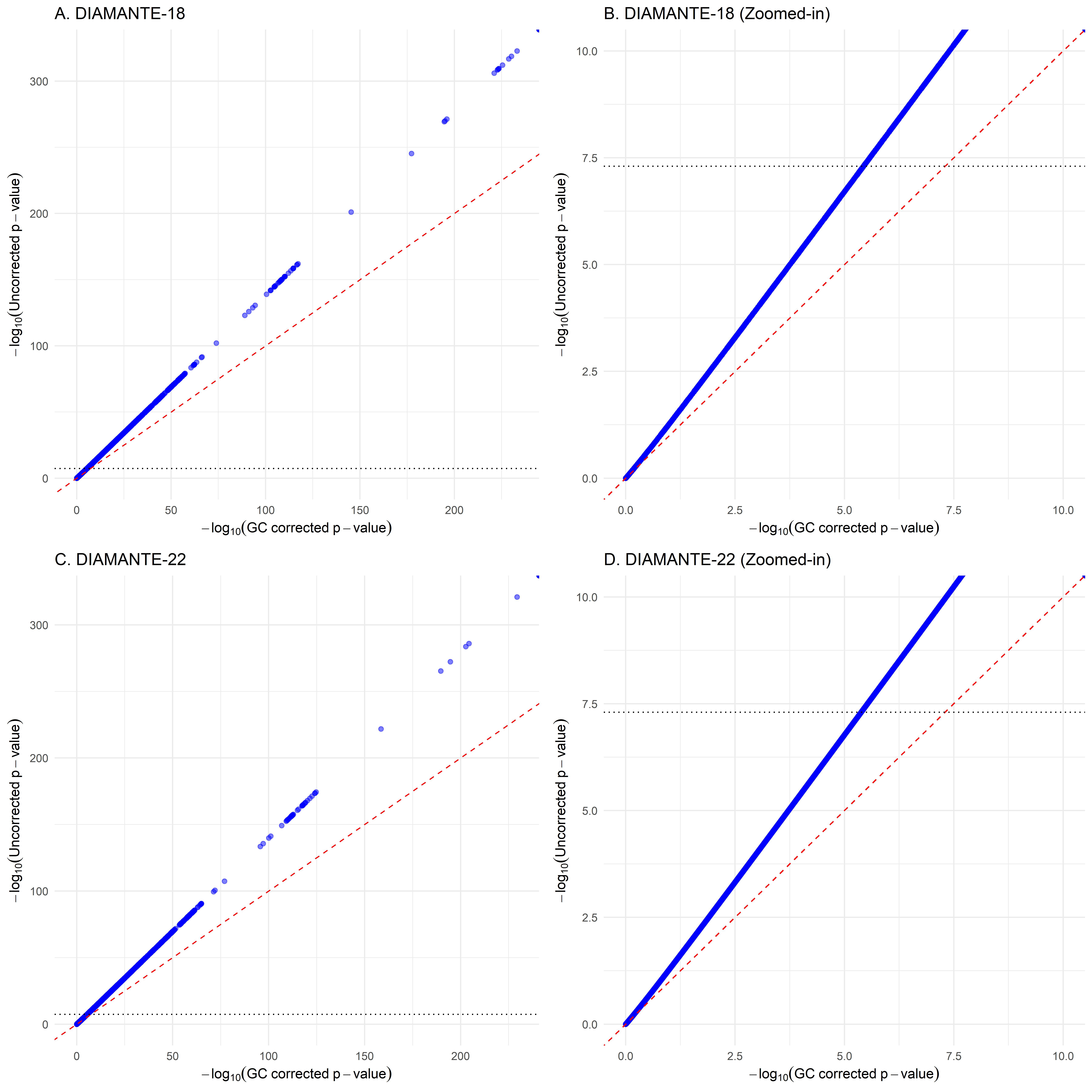


*The horizontal dotted line shows the genome-wide significance cutoff of* *5 × 10^-8^.*

Supplementary figure 5: Correlation plot showing p-values of SNPs across the genome before and after LDSR intercept correction in the DIAMANTE studies. **A**: DIAMANTE-18 and **B**: DIAMANTE-18: Zoomed-in around the genome-wide significance cutoff. **C**: DIAMANTE-22 and **D**: DIAMANTE-22: Zoomed-in around the genome-wide significance cutoff.


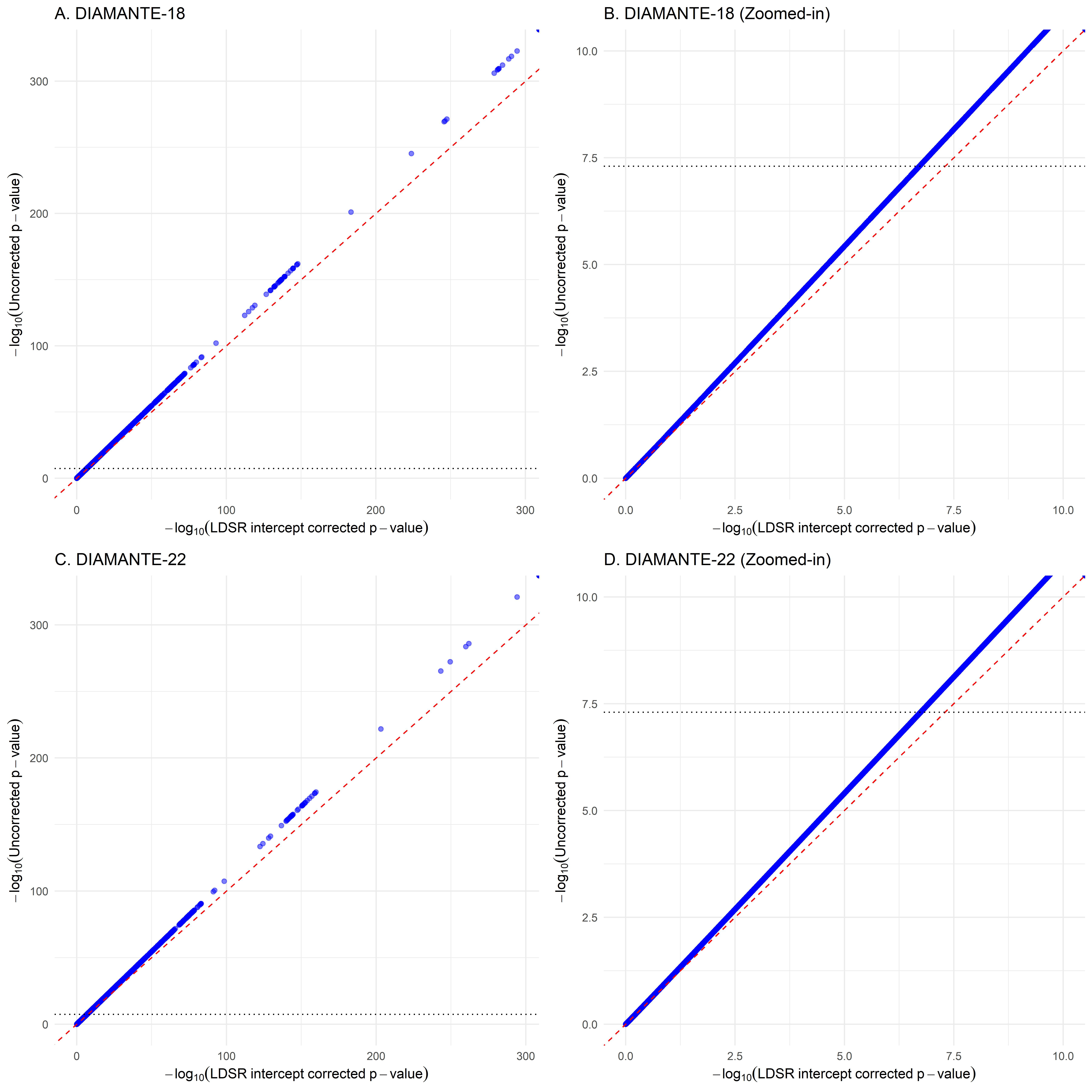


*The horizontal dotted line shows the genome-wide significance cutoff of 5 × 10^-8^.*

Supplementary figure 6: Comparing data distribution between robust associations lost only to GC and those lost to GC and LDSR intercept correction in the DIAMANTE-18 and the T2DGGI-24 analysis.

*P-value distribution: t-test p-value <* *2 × 10^-16^*

*Minor allele frequency distribution: t-test p-value < 2 × 10^-16^*

*LD score distribution: t-test p-value = 0.746*

Supplementary figure 7: Comparing data distribution between robust associations lost only to GC and those lost to GC and LDSR intercept correction in the DIAMANTE-22 and the T2DGGI-24 analysis.

*P-value distribution: t-test p-value < 2 × 10^-16^*

*Minor allele frequency distribution: t-test p-value = 0.10*

*LD score distribution: t-test p-value < 2 × 10^-7^*

**List of Type 2 Diabetes Global Genomics Initiative (T2DGGI) members**

Ken Suzuki[^1,^](https://diagram-consortium.org/T2DGGI.html#1)[^2,^](https://diagram-consortium.org/T2DGGI.html#2)[^3^](https://diagram-consortium.org/T2DGGI.html#3), Konstantinos Hatzikotoulas[^4^](https://diagram-consortium.org/T2DGGI.html#4), Lorraine Southam[^4^](https://diagram-consortium.org/T2DGGI.html#4), Henry J. Taylor[^5,^](https://diagram-consortium.org/T2DGGI.html#5)[^6,^](https://diagram-consortium.org/T2DGGI.html#6)[^7^](https://diagram-consortium.org/T2DGGI.html#7), Xianyong Yin[^8,^](https://diagram-consortium.org/T2DGGI.html#8)[^9^](https://diagram-consortium.org/T2DGGI.html#9), Kim M. Lorenz[^10,^](https://diagram-consortium.org/T2DGGI.html#10)[^11,^](https://diagram-consortium.org/T2DGGI.html#11)[^12^](https://diagram-consortium.org/T2DGGI.html#12), Ravi Mandla[^13,^](https://diagram-consortium.org/T2DGGI.html#13)[^14^](https://diagram-consortium.org/T2DGGI.html#14), Alicia Huerta-Chagoya[^13^](https://diagram-consortium.org/T2DGGI.html#13), Giorgio EM Melloni[^15^](https://diagram-consortium.org/T2DGGI.html#15), Stavroula Kanoni[^16^](https://diagram-consortium.org/T2DGGI.html#16), Nigel W Rayner[^4^](https://diagram-consortium.org/T2DGGI.html#4), Ozvan Bocher[^4^](https://diagram-consortium.org/T2DGGI.html#4), Ana Luiza Arruda[^4,^](https://diagram-consortium.org/T2DGGI.html#4)[^297,^](https://diagram-consortium.org/T2DGGI.html#297)[^298^](https://diagram-consortium.org/T2DGGI.html#298), Kyuto Sonehara[^17,^](https://diagram-consortium.org/T2DGGI.html#17)[^3,^](https://diagram-consortium.org/T2DGGI.html#3)[^18,^](https://diagram-consortium.org/T2DGGI.html#18)[^19^](https://diagram-consortium.org/T2DGGI.html#19), Shinichi Namba[^3^](https://diagram-consortium.org/T2DGGI.html#3), Simon SK Lee[^20^](https://diagram-consortium.org/T2DGGI.html#20), Michael H Preuss[^20^](https://diagram-consortium.org/T2DGGI.html#20), Lauren E Petty[^21^](https://diagram-consortium.org/T2DGGI.html#21), Philip Schroeder[^13,^](https://diagram-consortium.org/T2DGGI.html#13)[^14^](https://diagram-consortium.org/T2DGGI.html#14), Brett Vanderwerff[^9^](https://diagram-consortium.org/T2DGGI.html#9), Mart Kals[^22^](https://diagram-consortium.org/T2DGGI.html#22), Fiona Bragg[^23,^](https://diagram-consortium.org/T2DGGI.html#23)[^24^](https://diagram-consortium.org/T2DGGI.html#24), Kuang Lin[^23^](https://diagram-consortium.org/T2DGGI.html#23), Xiuqing Guo[^25^](https://diagram-consortium.org/T2DGGI.html#25), Weihua Zhang[^26,^](https://diagram-consortium.org/T2DGGI.html#26)[^27^](https://diagram-consortium.org/T2DGGI.html#27), Jie Yao[^25^](https://diagram-consortium.org/T2DGGI.html#25), Young Jin Kim[^28^](https://diagram-consortium.org/T2DGGI.html#28), Mariaelisa Graff[^29^](https://diagram-consortium.org/T2DGGI.html#29), Fumihiko Takeuchi[^30^](https://diagram-consortium.org/T2DGGI.html#30), Jana Nano[^31^](https://diagram-consortium.org/T2DGGI.html#31), Amel Lamri[^32,^](https://diagram-consortium.org/T2DGGI.html#32)[^33^](https://diagram-consortium.org/T2DGGI.html#33), Masahiro Nakatochi[^34^](https://diagram-consortium.org/T2DGGI.html#34), Sanghoon Moon[^28^](https://diagram-consortium.org/T2DGGI.html#28), Robert A Scott[^35^](https://diagram-consortium.org/T2DGGI.html#35), James P Cook[^36^](https://diagram-consortium.org/T2DGGI.html#36), Jung-Jin Lee[^37^](https://diagram-consortium.org/T2DGGI.html#37), Ian Pan[^38^](https://diagram-consortium.org/T2DGGI.html#38), Daniel Taliun[^9^](https://diagram-consortium.org/T2DGGI.html#9), Esteban J Parra[^39^](https://diagram-consortium.org/T2DGGI.html#39), Jin-Fang Chai[^40^](https://diagram-consortium.org/T2DGGI.html#40), Lawrence F Bielak[^41^](https://diagram-consortium.org/T2DGGI.html#41), Yasuharu Tabara[^42^](https://diagram-consortium.org/T2DGGI.html#42), Yang Hai[^25^](https://diagram-consortium.org/T2DGGI.html#25), Gudmar Thorleifsson[^43^](https://diagram-consortium.org/T2DGGI.html#43), Niels Grarup[^44^](https://diagram-consortium.org/T2DGGI.html#44), Tamar Sofer[^45,^](https://diagram-consortium.org/T2DGGI.html#45)[^46,^](https://diagram-consortium.org/T2DGGI.html#46)[^47^](https://diagram-consortium.org/T2DGGI.html#47), Matthias Wuttke[^48^](https://diagram-consortium.org/T2DGGI.html#48), Chloé Sarnowski[^49^](https://diagram-consortium.org/T2DGGI.html#49), Christian Gieger[^50,^](https://diagram-consortium.org/T2DGGI.html#50)[^31,^](https://diagram-consortium.org/T2DGGI.html#31)[^51^](https://diagram-consortium.org/T2DGGI.html#51), Darryl Nousome[^52^](https://diagram-consortium.org/T2DGGI.html#52), Stella Trompet[^53,^](https://diagram-consortium.org/T2DGGI.html#53)[^54^](https://diagram-consortium.org/T2DGGI.html#54), Soo-Heon Kwak[^55^](https://diagram-consortium.org/T2DGGI.html#55), Jirong Long[^56^](https://diagram-consortium.org/T2DGGI.html#56), Meng Sun[^57^](https://diagram-consortium.org/T2DGGI.html#57), Lin Tong[^58^](https://diagram-consortium.org/T2DGGI.html#58), Wei-Min Chen[^59^](https://diagram-consortium.org/T2DGGI.html#59), Suraj S Nongmaithem[^60^](https://diagram-consortium.org/T2DGGI.html#60), Raymond Noordam[^54^](https://diagram-consortium.org/T2DGGI.html#54), Victor JY Lim[^40^](https://diagram-consortium.org/T2DGGI.html#40), Claudia HT Tam[^61,^](https://diagram-consortium.org/T2DGGI.html#61)[^62^](https://diagram-consortium.org/T2DGGI.html#62), Yoonjung Yoonie Joo[^63,^](https://diagram-consortium.org/T2DGGI.html#63)[^64^](https://diagram-consortium.org/T2DGGI.html#64), Chien-Hsiun Chen[^65^](https://diagram-consortium.org/T2DGGI.html#65), Laura M Raffield[^66^](https://diagram-consortium.org/T2DGGI.html#66), Bram Peter Prins[^67^](https://diagram-consortium.org/T2DGGI.html#67), Aude Nicolas[^68^](https://diagram-consortium.org/T2DGGI.html#68), Lisa R Yanek[^69^](https://diagram-consortium.org/T2DGGI.html#69), Guanjie Chen[^70^](https://diagram-consortium.org/T2DGGI.html#70), Jennifer A Brody[^71^](https://diagram-consortium.org/T2DGGI.html#71), Edmond Kabagambe[^72,^](https://diagram-consortium.org/T2DGGI.html#72)[^56^](https://diagram-consortium.org/T2DGGI.html#56), Ping An[^73^](https://diagram-consortium.org/T2DGGI.html#73), Anny H Xiang[^74^](https://diagram-consortium.org/T2DGGI.html#74), Hyeok Sun Choi[^75^](https://diagram-consortium.org/T2DGGI.html#75), Brian E Cade[^46,^](https://diagram-consortium.org/T2DGGI.html#46)[^76^](https://diagram-consortium.org/T2DGGI.html#76), Jingyi Tan[^25^](https://diagram-consortium.org/T2DGGI.html#25), K Alaine Broadaway[^66^](https://diagram-consortium.org/T2DGGI.html#66), Alice Williamson[^35,^](https://diagram-consortium.org/T2DGGI.html#35)[^77^](https://diagram-consortium.org/T2DGGI.html#77), Zoha Kamali[^78,^](https://diagram-consortium.org/T2DGGI.html#78)[^79^](https://diagram-consortium.org/T2DGGI.html#79), Jinrui Cui[^80^](https://diagram-consortium.org/T2DGGI.html#80), Manonanthini Thangam[^81^](https://diagram-consortium.org/T2DGGI.html#81), Linda S Adair[^82^](https://diagram-consortium.org/T2DGGI.html#82), Adebowale Adeyemo[^70^](https://diagram-consortium.org/T2DGGI.html#70), Carlos A Aguilar-Salinas[^83^](https://diagram-consortium.org/T2DGGI.html#83), Tarunveer S Ahluwalia[^84,^](https://diagram-consortium.org/T2DGGI.html#84)[^85^](https://diagram-consortium.org/T2DGGI.html#85), Sonia S Anand[^32,^](https://diagram-consortium.org/T2DGGI.html#32)[^33,^](https://diagram-consortium.org/T2DGGI.html#33)[^86^](https://diagram-consortium.org/T2DGGI.html#86), Alain Bertoni[^87^](https://diagram-consortium.org/T2DGGI.html#87), Jette Bork-Jensen[^44^](https://diagram-consortium.org/T2DGGI.html#44), Ivan Brandslund[^88,^](https://diagram-consortium.org/T2DGGI.html#88)[^89^](https://diagram-consortium.org/T2DGGI.html#89), Thomas A Buchanan[^90^](https://diagram-consortium.org/T2DGGI.html#90), Charles F Burant[^91^](https://diagram-consortium.org/T2DGGI.html#91), Adam S Butterworth[^6,^](https://diagram-consortium.org/T2DGGI.html#6)[^92,^](https://diagram-consortium.org/T2DGGI.html#92)[^93,^](https://diagram-consortium.org/T2DGGI.html#93)[^7,^](https://diagram-consortium.org/T2DGGI.html#7)[^94^](https://diagram-consortium.org/T2DGGI.html#94), Mickaël Canouil[^95,^](https://diagram-consortium.org/T2DGGI.html#95)[^96^](https://diagram-consortium.org/T2DGGI.html#96), Juliana CN Chan[^61,^](https://diagram-consortium.org/T2DGGI.html#61)[^62,^](https://diagram-consortium.org/T2DGGI.html#62)[^97,^](https://diagram-consortium.org/T2DGGI.html#97)[^98^](https://diagram-consortium.org/T2DGGI.html#98), Li-Ching Chang[^65^](https://diagram-consortium.org/T2DGGI.html#65), Miao-Li Chee[^99^](https://diagram-consortium.org/T2DGGI.html#99), Ji Chen[^100,^](https://diagram-consortium.org/T2DGGI.html#100)[^101^](https://diagram-consortium.org/T2DGGI.html#101), Shyh-Huei Chen[^102^](https://diagram-consortium.org/T2DGGI.html#102), Yuan-Tsong Chen[^65^](https://diagram-consortium.org/T2DGGI.html#65), Zhengming Chen[^23,^](https://diagram-consortium.org/T2DGGI.html#23)[^24^](https://diagram-consortium.org/T2DGGI.html#24), Lee-Ming Chuang[^103,^](https://diagram-consortium.org/T2DGGI.html#103)[^104^](https://diagram-consortium.org/T2DGGI.html#104), Mary Cushman[^105^](https://diagram-consortium.org/T2DGGI.html#105), John Danesh[^6,^](https://diagram-consortium.org/T2DGGI.html#6)[^92,^](https://diagram-consortium.org/T2DGGI.html#92)[^93,^](https://diagram-consortium.org/T2DGGI.html#93)[^67,^](https://diagram-consortium.org/T2DGGI.html#67)[^7,^](https://diagram-consortium.org/T2DGGI.html#7)[^94^](https://diagram-consortium.org/T2DGGI.html#94), Swapan K Das[^106^](https://diagram-consortium.org/T2DGGI.html#106), H Janaka de Silva[^107^](https://diagram-consortium.org/T2DGGI.html#107), George Dedoussis[^108^](https://diagram-consortium.org/T2DGGI.html#108), Latchezar Dimitrov[^109^](https://diagram-consortium.org/T2DGGI.html#109), Ayo P Doumatey[^70^](https://diagram-consortium.org/T2DGGI.html#70), Shufa Du[^82,^](https://diagram-consortium.org/T2DGGI.html#82)[^110^](https://diagram-consortium.org/T2DGGI.html#110), Qing Duan[^66^](https://diagram-consortium.org/T2DGGI.html#66), Kai-Uwe Eckardt[^111,^](https://diagram-consortium.org/T2DGGI.html#111)[^112^](https://diagram-consortium.org/T2DGGI.html#112), Leslie S Emery[^113^](https://diagram-consortium.org/T2DGGI.html#113), Daniel S Evans[^114^](https://diagram-consortium.org/T2DGGI.html#114), Michele K Evans[^115^](https://diagram-consortium.org/T2DGGI.html#115), Krista Fischer[^22,^](https://diagram-consortium.org/T2DGGI.html#22)[^116^](https://diagram-consortium.org/T2DGGI.html#116), James S Floyd[^71^](https://diagram-consortium.org/T2DGGI.html#71), Ian Ford[^117^](https://diagram-consortium.org/T2DGGI.html#117), Oscar H Franco[^118^](https://diagram-consortium.org/T2DGGI.html#118), Timothy M Frayling[^119^](https://diagram-consortium.org/T2DGGI.html#119), Barry I Freedman[^120^](https://diagram-consortium.org/T2DGGI.html#120), Pauline Genter[^121^](https://diagram-consortium.org/T2DGGI.html#121), Hertzel C Gerstein[^32,^](https://diagram-consortium.org/T2DGGI.html#32)[^33,^](https://diagram-consortium.org/T2DGGI.html#33)[^86^](https://diagram-consortium.org/T2DGGI.html#86), Vilmantas Giedraitis[^122^](https://diagram-consortium.org/T2DGGI.html#122), Clicerio González-Villalpando[^123^](https://diagram-consortium.org/T2DGGI.html#123), Maria Elena González-Villalpando[^123^](https://diagram-consortium.org/T2DGGI.html#123), Penny Gordon-Larsen[^82,^](https://diagram-consortium.org/T2DGGI.html#82)[^110^](https://diagram-consortium.org/T2DGGI.html#110), Myron Gross[^124^](https://diagram-consortium.org/T2DGGI.html#124), Lindsay A Guare[^125^](https://diagram-consortium.org/T2DGGI.html#125), Sophie Hackinger[^67^](https://diagram-consortium.org/T2DGGI.html#67), Liisa Hakaste[^126,^](https://diagram-consortium.org/T2DGGI.html#126)[^127^](https://diagram-consortium.org/T2DGGI.html#127), Sohee Han[^28^](https://diagram-consortium.org/T2DGGI.html#28), Andrew T Hattersley[^128^](https://diagram-consortium.org/T2DGGI.html#128), Christian Herder[^50,^](https://diagram-consortium.org/T2DGGI.html#50)[^129,^](https://diagram-consortium.org/T2DGGI.html#129)[^130^](https://diagram-consortium.org/T2DGGI.html#130), Momoko Horikoshi[^131^](https://diagram-consortium.org/T2DGGI.html#131), Annie-Green Howard[^132,^](https://diagram-consortium.org/T2DGGI.html#132)[^110^](https://diagram-consortium.org/T2DGGI.html#110), Willa Hsueh[^133^](https://diagram-consortium.org/T2DGGI.html#133), Mengna Huang[^38,^](https://diagram-consortium.org/T2DGGI.html#38)[^134^](https://diagram-consortium.org/T2DGGI.html#134), Wei Huang[^135^](https://diagram-consortium.org/T2DGGI.html#135), Yi-Jen Hung[^136,^](https://diagram-consortium.org/T2DGGI.html#136)[^137^](https://diagram-consortium.org/T2DGGI.html#137), Mi Yeong Hwang[^138^](https://diagram-consortium.org/T2DGGI.html#138), Chii-Min Hwu[^139,^](https://diagram-consortium.org/T2DGGI.html#139)[^140^](https://diagram-consortium.org/T2DGGI.html#140), Sahoko Ichihara[^141^](https://diagram-consortium.org/T2DGGI.html#141), Mohammad Arfan Ikram[^118^](https://diagram-consortium.org/T2DGGI.html#118), Martin Ingelsson[^122^](https://diagram-consortium.org/T2DGGI.html#122), Md. Tariqul Islam[^142^](https://diagram-consortium.org/T2DGGI.html#142), Masato Isono[^30^](https://diagram-consortium.org/T2DGGI.html#30), Hye-Mi Jang[^138^](https://diagram-consortium.org/T2DGGI.html#138), Farzana Jasmine[^58^](https://diagram-consortium.org/T2DGGI.html#58), Guozhi Jiang[^61,^](https://diagram-consortium.org/T2DGGI.html#61)[^62^](https://diagram-consortium.org/T2DGGI.html#62), Jost B Jonas[^143^](https://diagram-consortium.org/T2DGGI.html#143), Torben Jørgensen[^144,^](https://diagram-consortium.org/T2DGGI.html#144)[^145,^](https://diagram-consortium.org/T2DGGI.html#145)[^146^](https://diagram-consortium.org/T2DGGI.html#146), Frederick K Kamanu[^15^](https://diagram-consortium.org/T2DGGI.html#15), Fouad R Kandeel[^147^](https://diagram-consortium.org/T2DGGI.html#147), Anuradhani Kasturiratne[^148^](https://diagram-consortium.org/T2DGGI.html#148), Tomohiro Katsuya[^149,^](https://diagram-consortium.org/T2DGGI.html#149)[^150^](https://diagram-consortium.org/T2DGGI.html#150), Varinderpal Kaur[^14^](https://diagram-consortium.org/T2DGGI.html#14), Takahisa Kawaguchi[^42^](https://diagram-consortium.org/T2DGGI.html#42), Jacob M Keaton[^5,^](https://diagram-consortium.org/T2DGGI.html#5)[^56,^](https://diagram-consortium.org/T2DGGI.html#56)[^109^](https://diagram-consortium.org/T2DGGI.html#109), Abel N Kho[^151,^](https://diagram-consortium.org/T2DGGI.html#151)[^152^](https://diagram-consortium.org/T2DGGI.html#152), Chiea-Chuen Khor[^153^](https://diagram-consortium.org/T2DGGI.html#153), Muhammad G Kibriya[^58^](https://diagram-consortium.org/T2DGGI.html#58), Duk-Hwan Kim[^154^](https://diagram-consortium.org/T2DGGI.html#154), Florian Kronenberg[^155^](https://diagram-consortium.org/T2DGGI.html#155), Johanna Kuusisto[^156^](https://diagram-consortium.org/T2DGGI.html#156), Kristi Läll[^22^](https://diagram-consortium.org/T2DGGI.html#22), Leslie A Lange[^157^](https://diagram-consortium.org/T2DGGI.html#157), Kyung Min Lee[^158,^](https://diagram-consortium.org/T2DGGI.html#158)[^159^](https://diagram-consortium.org/T2DGGI.html#159), Myung-Shik Lee[^160,^](https://diagram-consortium.org/T2DGGI.html#160)[^161^](https://diagram-consortium.org/T2DGGI.html#161), Nanette R Lee[^162^](https://diagram-consortium.org/T2DGGI.html#162), Aaron Leong[^163,^](https://diagram-consortium.org/T2DGGI.html#163)[^164^](https://diagram-consortium.org/T2DGGI.html#164), Liming Li[^165,^](https://diagram-consortium.org/T2DGGI.html#165)[^166^](https://diagram-consortium.org/T2DGGI.html#166), Yun Li[^66^](https://diagram-consortium.org/T2DGGI.html#66), Ruifang Li-Gao[^167^](https://diagram-consortium.org/T2DGGI.html#167), Symen Ligthart[^118^](https://diagram-consortium.org/T2DGGI.html#118), Cecilia M Lindgren[^168,^](https://diagram-consortium.org/T2DGGI.html#168)[^169,^](https://diagram-consortium.org/T2DGGI.html#169)[^170^](https://diagram-consortium.org/T2DGGI.html#170), Allan Linneberg[^144,^](https://diagram-consortium.org/T2DGGI.html#144)[^171^](https://diagram-consortium.org/T2DGGI.html#171), Ching-Ti Liu[^172^](https://diagram-consortium.org/T2DGGI.html#172), Jianjun Liu[^153,^](https://diagram-consortium.org/T2DGGI.html#153)[^173^](https://diagram-consortium.org/T2DGGI.html#173), Adam E Locke[^174,^](https://diagram-consortium.org/T2DGGI.html#174)[^175,^](https://diagram-consortium.org/T2DGGI.html#175)[^302^](https://diagram-consortium.org/T2DGGI.html#302), Tin Louie[^113^](https://diagram-consortium.org/T2DGGI.html#113), Jian'an Luan[^35^](https://diagram-consortium.org/T2DGGI.html#35), Andrea O Luk[^61,^](https://diagram-consortium.org/T2DGGI.html#61)[^62^](https://diagram-consortium.org/T2DGGI.html#62), Xi Luo[^176^](https://diagram-consortium.org/T2DGGI.html#176), Jun Lv[^165,^](https://diagram-consortium.org/T2DGGI.html#165)[^166^](https://diagram-consortium.org/T2DGGI.html#166), Julie A Lynch[^158,^](https://diagram-consortium.org/T2DGGI.html#158)[^159^](https://diagram-consortium.org/T2DGGI.html#159), Valeriya Lyssenko[^177,^](https://diagram-consortium.org/T2DGGI.html#177)[^178^](https://diagram-consortium.org/T2DGGI.html#178), Shiro Maeda[^179,^](https://diagram-consortium.org/T2DGGI.html#179)[^180,^](https://diagram-consortium.org/T2DGGI.html#180)[^131^](https://diagram-consortium.org/T2DGGI.html#131), Vasiliki Mamakou[^181^](https://diagram-consortium.org/T2DGGI.html#181), Sohail Rafik Mansuri[^60,^](https://diagram-consortium.org/T2DGGI.html#60)[^299^](https://diagram-consortium.org/T2DGGI.html#299), Koichi Matsuda[^182^](https://diagram-consortium.org/T2DGGI.html#182), Thomas Meitinger[^183,^](https://diagram-consortium.org/T2DGGI.html#183)[^184,^](https://diagram-consortium.org/T2DGGI.html#184)[^185^](https://diagram-consortium.org/T2DGGI.html#185), Olle Melander[^81^](https://diagram-consortium.org/T2DGGI.html#81), Andres Metspalu[^22^](https://diagram-consortium.org/T2DGGI.html#22), Huan Mo[^5^](https://diagram-consortium.org/T2DGGI.html#5), Andrew D Morris[^186^](https://diagram-consortium.org/T2DGGI.html#186), Filipe A Moura[^15^](https://diagram-consortium.org/T2DGGI.html#15), Jerry L Nadler[^187^](https://diagram-consortium.org/T2DGGI.html#187), Michael A Nalls[^68,^](https://diagram-consortium.org/T2DGGI.html#68)[^188,^](https://diagram-consortium.org/T2DGGI.html#188)[^189^](https://diagram-consortium.org/T2DGGI.html#189), Uma Nayak[^59^](https://diagram-consortium.org/T2DGGI.html#59), Ioanna Ntalla[^16^](https://diagram-consortium.org/T2DGGI.html#16), Yukinori Okada[^3,^](https://diagram-consortium.org/T2DGGI.html#3)[^17,^](https://diagram-consortium.org/T2DGGI.html#17)[^19,^](https://diagram-consortium.org/T2DGGI.html#19)[^190,^](https://diagram-consortium.org/T2DGGI.html#190)[^18,^](https://diagram-consortium.org/T2DGGI.html#18)[^191^](https://diagram-consortium.org/T2DGGI.html#191), Lorena Orozco[^192^](https://diagram-consortium.org/T2DGGI.html#192), Sanjay R Patel[^193^](https://diagram-consortium.org/T2DGGI.html#193), Snehal Patil[^9^](https://diagram-consortium.org/T2DGGI.html#9), Pei Pei[^166^](https://diagram-consortium.org/T2DGGI.html#166), Mark A Pereira[^194^](https://diagram-consortium.org/T2DGGI.html#194), Annette Peters[^50,^](https://diagram-consortium.org/T2DGGI.html#50)[^185,^](https://diagram-consortium.org/T2DGGI.html#185)[^31,^](https://diagram-consortium.org/T2DGGI.html#31)[^195^](https://diagram-consortium.org/T2DGGI.html#195), Fraser J Pirie[^196^](https://diagram-consortium.org/T2DGGI.html#196), Hannah G Polikowsky[^21^](https://diagram-consortium.org/T2DGGI.html#21), Bianca Porneala[^164^](https://diagram-consortium.org/T2DGGI.html#164), Gauri Prasad[^197,^](https://diagram-consortium.org/T2DGGI.html#197)[^198^](https://diagram-consortium.org/T2DGGI.html#198), Laura J Rasmussen-Torvik[^199^](https://diagram-consortium.org/T2DGGI.html#199), Alexander P Reiner[^200^](https://diagram-consortium.org/T2DGGI.html#200), Michael Roden[^50,^](https://diagram-consortium.org/T2DGGI.html#50)[^129,^](https://diagram-consortium.org/T2DGGI.html#129)[^130^](https://diagram-consortium.org/T2DGGI.html#130), Rebecca Rohde[^29^](https://diagram-consortium.org/T2DGGI.html#29), Katheryn Roll[^25^](https://diagram-consortium.org/T2DGGI.html#25), Charumathi Sabanayagam[^99,^](https://diagram-consortium.org/T2DGGI.html#99)[^201,^](https://diagram-consortium.org/T2DGGI.html#201)[^202^](https://diagram-consortium.org/T2DGGI.html#202), Kevin Sandow[^25^](https://diagram-consortium.org/T2DGGI.html#25), Alagu Sankareswaran[^60,^](https://diagram-consortium.org/T2DGGI.html#60)[^299^](https://diagram-consortium.org/T2DGGI.html#299), Naveed Sattar[^203^](https://diagram-consortium.org/T2DGGI.html#203), Sebastian Schönherr[^155^](https://diagram-consortium.org/T2DGGI.html#155), Mohammad Shahriar[^58^](https://diagram-consortium.org/T2DGGI.html#58), Botong Shen[^115^](https://diagram-consortium.org/T2DGGI.html#115), Jinxiu Shi[^135^](https://diagram-consortium.org/T2DGGI.html#135), Dong Mun Shin[^138^](https://diagram-consortium.org/T2DGGI.html#138), Nobuhiro Shojima[^2^](https://diagram-consortium.org/T2DGGI.html#2), Jennifer A Smith[^41,^](https://diagram-consortium.org/T2DGGI.html#41)[^204^](https://diagram-consortium.org/T2DGGI.html#204), Wing Yee So[^61,^](https://diagram-consortium.org/T2DGGI.html#61)[^98^](https://diagram-consortium.org/T2DGGI.html#98), Alena Stančáková[^156^](https://diagram-consortium.org/T2DGGI.html#156), Valgerdur Steinthorsdottir[^43^](https://diagram-consortium.org/T2DGGI.html#43), Adrienne M Stilp[^113^](https://diagram-consortium.org/T2DGGI.html#113), Konstantin Strauch[^205,^](https://diagram-consortium.org/T2DGGI.html#205)[^206,^](https://diagram-consortium.org/T2DGGI.html#206)[^207^](https://diagram-consortium.org/T2DGGI.html#207), Kent D Taylor[^25^](https://diagram-consortium.org/T2DGGI.html#25), Barbara Thorand[^50,^](https://diagram-consortium.org/T2DGGI.html#50)[^31^](https://diagram-consortium.org/T2DGGI.html#31), Unnur Thorsteinsdottir[^43,^](https://diagram-consortium.org/T2DGGI.html#43)[^208^](https://diagram-consortium.org/T2DGGI.html#208), Brian Tomlinson[^61,^](https://diagram-consortium.org/T2DGGI.html#61)[^209^](https://diagram-consortium.org/T2DGGI.html#209), Tam C. Tran[^5^](https://diagram-consortium.org/T2DGGI.html#5), Fuu-Jen Tsai[^210^](https://diagram-consortium.org/T2DGGI.html#210), Jaakko Tuomilehto[^211,^](https://diagram-consortium.org/T2DGGI.html#211)[^212,^](https://diagram-consortium.org/T2DGGI.html#212)[^213,^](https://diagram-consortium.org/T2DGGI.html#213)[^214^](https://diagram-consortium.org/T2DGGI.html#214), Teresa Tusie-Luna[^215,^](https://diagram-consortium.org/T2DGGI.html#215)[^216^](https://diagram-consortium.org/T2DGGI.html#216), Miriam S Udler[^163,^](https://diagram-consortium.org/T2DGGI.html#163)[^13,^](https://diagram-consortium.org/T2DGGI.html#13)[^14^](https://diagram-consortium.org/T2DGGI.html#14), Adan Valladares-Salgado[^217^](https://diagram-consortium.org/T2DGGI.html#217), Rob M van Dam[^40,^](https://diagram-consortium.org/T2DGGI.html#40)[^173^](https://diagram-consortium.org/T2DGGI.html#173), Jan B van Klinken[^218,^](https://diagram-consortium.org/T2DGGI.html#218)[^219,^](https://diagram-consortium.org/T2DGGI.html#219)[^220^](https://diagram-consortium.org/T2DGGI.html#220), Rohit Varma[^221^](https://diagram-consortium.org/T2DGGI.html#221), Niels Wacher-Rodarte[^222^](https://diagram-consortium.org/T2DGGI.html#222), Eleanor Wheeler[^35^](https://diagram-consortium.org/T2DGGI.html#35), Ananda R Wickremasinghe[^148^](https://diagram-consortium.org/T2DGGI.html#148), Ko Willems van Dijk[^218,^](https://diagram-consortium.org/T2DGGI.html#218)[^219,^](https://diagram-consortium.org/T2DGGI.html#219)[^223^](https://diagram-consortium.org/T2DGGI.html#223), Daniel R Witte[^224,^](https://diagram-consortium.org/T2DGGI.html#224)[^225^](https://diagram-consortium.org/T2DGGI.html#225), Chittaranjan S Yajnik[^226^](https://diagram-consortium.org/T2DGGI.html#226), Ken Yamamoto[^227^](https://diagram-consortium.org/T2DGGI.html#227), Kenichi Yamamoto[^3,^](https://diagram-consortium.org/T2DGGI.html#3)[^228,^](https://diagram-consortium.org/T2DGGI.html#228)[^190^](https://diagram-consortium.org/T2DGGI.html#190), Kyungheon Yoon[^138^](https://diagram-consortium.org/T2DGGI.html#138), Canqing Yu[^165,^](https://diagram-consortium.org/T2DGGI.html#165)[^166^](https://diagram-consortium.org/T2DGGI.html#166), Jian-Min Yuan[^229,^](https://diagram-consortium.org/T2DGGI.html#229)[^230^](https://diagram-consortium.org/T2DGGI.html#230), Salim Yusuf[^33,^](https://diagram-consortium.org/T2DGGI.html#33)[^32,^](https://diagram-consortium.org/T2DGGI.html#32)[^86^](https://diagram-consortium.org/T2DGGI.html#86), Matthew Zawistowski[^9^](https://diagram-consortium.org/T2DGGI.html#9), Liang Zhang[^99^](https://diagram-consortium.org/T2DGGI.html#99), Wei Zheng[^56^](https://diagram-consortium.org/T2DGGI.html#56), Leslie J Raffel[^231^](https://diagram-consortium.org/T2DGGI.html#231), Michiya Igase[^232^](https://diagram-consortium.org/T2DGGI.html#232), Eli Ipp[^121^](https://diagram-consortium.org/T2DGGI.html#121), Susan Redline[^46,^](https://diagram-consortium.org/T2DGGI.html#46)[^233,^](https://diagram-consortium.org/T2DGGI.html#233)[^76^](https://diagram-consortium.org/T2DGGI.html#76), Yoon Shin Cho[^75^](https://diagram-consortium.org/T2DGGI.html#75), Lars Lind[^234^](https://diagram-consortium.org/T2DGGI.html#234), Michael A Province[^73^](https://diagram-consortium.org/T2DGGI.html#73), Myriam Fornage[^235^](https://diagram-consortium.org/T2DGGI.html#235), Craig L Hanis[^236^](https://diagram-consortium.org/T2DGGI.html#236), Erik Ingelsson[^237,^](https://diagram-consortium.org/T2DGGI.html#237)[^238^](https://diagram-consortium.org/T2DGGI.html#238), Alan B Zonderman[^115^](https://diagram-consortium.org/T2DGGI.html#115), Bruce M Psaty[^71,^](https://diagram-consortium.org/T2DGGI.html#71)[^239,^](https://diagram-consortium.org/T2DGGI.html#239)[^240^](https://diagram-consortium.org/T2DGGI.html#240), Ya-Xing Wang[^241^](https://diagram-consortium.org/T2DGGI.html#241), Charles N Rotimi[^70^](https://diagram-consortium.org/T2DGGI.html#70), Diane M Becker[^69^](https://diagram-consortium.org/T2DGGI.html#69), Fumihiko Matsuda[^42^](https://diagram-consortium.org/T2DGGI.html#42), Yongmei Liu[^87,^](https://diagram-consortium.org/T2DGGI.html#87)[^242^](https://diagram-consortium.org/T2DGGI.html#242), Mitsuhiro Yokota[^243^](https://diagram-consortium.org/T2DGGI.html#243), Sharon LR Kardia[^41^](https://diagram-consortium.org/T2DGGI.html#41), Patricia A Peyser[^41^](https://diagram-consortium.org/T2DGGI.html#41), James S Pankow[^194^](https://diagram-consortium.org/T2DGGI.html#194), James C Engert[^244,^](https://diagram-consortium.org/T2DGGI.html#244)[^245^](https://diagram-consortium.org/T2DGGI.html#245), Amélie Bonnefond[^95,^](https://diagram-consortium.org/T2DGGI.html#95)[^96,^](https://diagram-consortium.org/T2DGGI.html#96)[^246^](https://diagram-consortium.org/T2DGGI.html#246), Philippe Froguel[^95,^](https://diagram-consortium.org/T2DGGI.html#95)[^96,^](https://diagram-consortium.org/T2DGGI.html#96)[^246^](https://diagram-consortium.org/T2DGGI.html#246), James G Wilson[^247^](https://diagram-consortium.org/T2DGGI.html#247), Wayne HH Sheu[^248,^](https://diagram-consortium.org/T2DGGI.html#248)[^140,^](https://diagram-consortium.org/T2DGGI.html#140)[^137^](https://diagram-consortium.org/T2DGGI.html#137), Jer-Yuarn Wu[^65^](https://diagram-consortium.org/T2DGGI.html#65), M Geoffrey Hayes[^249,^](https://diagram-consortium.org/T2DGGI.html#249)[^250,^](https://diagram-consortium.org/T2DGGI.html#250)[^251^](https://diagram-consortium.org/T2DGGI.html#251), Ronald CW Ma[^61,^](https://diagram-consortium.org/T2DGGI.html#61)[^62,^](https://diagram-consortium.org/T2DGGI.html#62)[^97,^](https://diagram-consortium.org/T2DGGI.html#97)[^98^](https://diagram-consortium.org/T2DGGI.html#98), Tien-Yin Wong[^99,^](https://diagram-consortium.org/T2DGGI.html#99)[^201,^](https://diagram-consortium.org/T2DGGI.html#201)[^202^](https://diagram-consortium.org/T2DGGI.html#202), Dennis O Mook-Kanamori[^167^](https://diagram-consortium.org/T2DGGI.html#167), Tiinamaija Tuomi[^252,^](https://diagram-consortium.org/T2DGGI.html#252)[^126,^](https://diagram-consortium.org/T2DGGI.html#126)[^127,^](https://diagram-consortium.org/T2DGGI.html#127)[^81^](https://diagram-consortium.org/T2DGGI.html#81), Giriraj R Chandak[^60,^](https://diagram-consortium.org/T2DGGI.html#60)[^300^](https://diagram-consortium.org/T2DGGI.html#300), Francis S Collins[^5^](https://diagram-consortium.org/T2DGGI.html#5), Dwaipayan Bharadwaj[^253^](https://diagram-consortium.org/T2DGGI.html#253), Guillaume Paré[^254,^](https://diagram-consortium.org/T2DGGI.html#254)[^33^](https://diagram-consortium.org/T2DGGI.html#33), Michèle M Sale[^59^](https://diagram-consortium.org/T2DGGI.html#59), Habibul Ahsan[^58^](https://diagram-consortium.org/T2DGGI.html#58), Ayesha A Motala[^196^](https://diagram-consortium.org/T2DGGI.html#196), Xiao-Ou Shu[^56^](https://diagram-consortium.org/T2DGGI.html#56), Kyong-Soo Park[^55,^](https://diagram-consortium.org/T2DGGI.html#55)[^255^](https://diagram-consortium.org/T2DGGI.html#255), J Wouter Jukema[^53,^](https://diagram-consortium.org/T2DGGI.html#53)[^256^](https://diagram-consortium.org/T2DGGI.html#256), Miguel Cruz[^217^](https://diagram-consortium.org/T2DGGI.html#217), Yii-Der Ida Chen[^25^](https://diagram-consortium.org/T2DGGI.html#25), Stephen S Rich[^257^](https://diagram-consortium.org/T2DGGI.html#257), Roberta McKean-Cowdin[^52^](https://diagram-consortium.org/T2DGGI.html#52), Harald Grallert[^31,^](https://diagram-consortium.org/T2DGGI.html#31)[^50,^](https://diagram-consortium.org/T2DGGI.html#50)[^258^](https://diagram-consortium.org/T2DGGI.html#258), Ching-Yu Cheng[^99,^](https://diagram-consortium.org/T2DGGI.html#99)[^201,^](https://diagram-consortium.org/T2DGGI.html#201)[^202^](https://diagram-consortium.org/T2DGGI.html#202), Mohsen Ghanbari [^118^](https://diagram-consortium.org/T2DGGI.html#118), E-Shyong Tai[^173,^](https://diagram-consortium.org/T2DGGI.html#173)[^40,^](https://diagram-consortium.org/T2DGGI.html#40)[^259^](https://diagram-consortium.org/T2DGGI.html#259), Josee Dupuis[^260,^](https://diagram-consortium.org/T2DGGI.html#260)[^172^](https://diagram-consortium.org/T2DGGI.html#172), Norihiro Kato[^30^](https://diagram-consortium.org/T2DGGI.html#30), Markku Laakso[^156^](https://diagram-consortium.org/T2DGGI.html#156), Anna Köttgen[^48^](https://diagram-consortium.org/T2DGGI.html#48), Woon-Puay Koh[^261,^](https://diagram-consortium.org/T2DGGI.html#261)[^262^](https://diagram-consortium.org/T2DGGI.html#262), Donald W Bowden[^109,^](https://diagram-consortium.org/T2DGGI.html#109)[^263,^](https://diagram-consortium.org/T2DGGI.html#263)[^264^](https://diagram-consortium.org/T2DGGI.html#264), Colin NA Palmer[^265^](https://diagram-consortium.org/T2DGGI.html#265), Jaspal S Kooner[^27,^](https://diagram-consortium.org/T2DGGI.html#27)[^266,^](https://diagram-consortium.org/T2DGGI.html#266)[^267,^](https://diagram-consortium.org/T2DGGI.html#267)[^268^](https://diagram-consortium.org/T2DGGI.html#268), Charles Kooperberg[^200^](https://diagram-consortium.org/T2DGGI.html#200), Simin Liu[^38,^](https://diagram-consortium.org/T2DGGI.html#38)[^134,^](https://diagram-consortium.org/T2DGGI.html#134)[^269^](https://diagram-consortium.org/T2DGGI.html#269), Kari E North[^29^](https://diagram-consortium.org/T2DGGI.html#29), Danish Saleheen[^270,^](https://diagram-consortium.org/T2DGGI.html#270)[^271,^](https://diagram-consortium.org/T2DGGI.html#271)[^272^](https://diagram-consortium.org/T2DGGI.html#272), Torben Hansen[^44^](https://diagram-consortium.org/T2DGGI.html#44), Oluf Pedersen[^44^](https://diagram-consortium.org/T2DGGI.html#44), Nicholas J Wareham[^35^](https://diagram-consortium.org/T2DGGI.html#35), Juyoung Lee[^138^](https://diagram-consortium.org/T2DGGI.html#138), Bong-Jo Kim[^138^](https://diagram-consortium.org/T2DGGI.html#138), Iona Y Millwood[^23,^](https://diagram-consortium.org/T2DGGI.html#23)[^24^](https://diagram-consortium.org/T2DGGI.html#24), Robin G Walters[^23,^](https://diagram-consortium.org/T2DGGI.html#23)[^24^](https://diagram-consortium.org/T2DGGI.html#24), Kari Stefansson[^43,^](https://diagram-consortium.org/T2DGGI.html#43)[^208^](https://diagram-consortium.org/T2DGGI.html#208), Emma Ahlqvist[^81^](https://diagram-consortium.org/T2DGGI.html#81), Mark O Goodarzi[^80^](https://diagram-consortium.org/T2DGGI.html#80), Karen L Mohlke[^66^](https://diagram-consortium.org/T2DGGI.html#66), Claudia Langenberg[^35,^](https://diagram-consortium.org/T2DGGI.html#35)[^273,^](https://diagram-consortium.org/T2DGGI.html#273)[^274^](https://diagram-consortium.org/T2DGGI.html#274), Christopher A Haiman[^275^](https://diagram-consortium.org/T2DGGI.html#275), Ruth JF Loos[^20,^](https://diagram-consortium.org/T2DGGI.html#20)[^276,^](https://diagram-consortium.org/T2DGGI.html#276)[^44^](https://diagram-consortium.org/T2DGGI.html#44), Jose C Florez[^163,^](https://diagram-consortium.org/T2DGGI.html#163)[^13,^](https://diagram-consortium.org/T2DGGI.html#13)[^14^](https://diagram-consortium.org/T2DGGI.html#14), Daniel J Rader[^277,^](https://diagram-consortium.org/T2DGGI.html#277)[^12,^](https://diagram-consortium.org/T2DGGI.html#12)[^278,^](https://diagram-consortium.org/T2DGGI.html#278)[^279^](https://diagram-consortium.org/T2DGGI.html#279), Marylyn D Ritchie[^280,^](https://diagram-consortium.org/T2DGGI.html#280)[^12,^](https://diagram-consortium.org/T2DGGI.html#12)[^281^](https://diagram-consortium.org/T2DGGI.html#281), Sebastian Zöllner[^9,^](https://diagram-consortium.org/T2DGGI.html#9)[^282^](https://diagram-consortium.org/T2DGGI.html#282), Reedik Mägi[^22^](https://diagram-consortium.org/T2DGGI.html#22), Nicholas A Marston[^15^](https://diagram-consortium.org/T2DGGI.html#15), Christian T Ruff[^15^](https://diagram-consortium.org/T2DGGI.html#15), David A van Heel[^283^](https://diagram-consortium.org/T2DGGI.html#283), Sarah Finer[^284^](https://diagram-consortium.org/T2DGGI.html#284), Joshua C Denny[^5,^](https://diagram-consortium.org/T2DGGI.html#5)[^285^](https://diagram-consortium.org/T2DGGI.html#285), Toshimasa Yamauchi[^2^](https://diagram-consortium.org/T2DGGI.html#2), Takashi Kadowaki[^2,^](https://diagram-consortium.org/T2DGGI.html#2)[^286^](https://diagram-consortium.org/T2DGGI.html#286), John C Chambers[^287,^](https://diagram-consortium.org/T2DGGI.html#287)[^26,^](https://diagram-consortium.org/T2DGGI.html#26)[^27,^](https://diagram-consortium.org/T2DGGI.html#27)[^266^](https://diagram-consortium.org/T2DGGI.html#266), Maggie CY Ng[^288,^](https://diagram-consortium.org/T2DGGI.html#288)[^109,^](https://diagram-consortium.org/T2DGGI.html#109)[^264^](https://diagram-consortium.org/T2DGGI.html#264), Xueling Sim[^40^](https://diagram-consortium.org/T2DGGI.html#40), Jennifer E Below[^21^](https://diagram-consortium.org/T2DGGI.html#21), Philip S Tsao[^289,^](https://diagram-consortium.org/T2DGGI.html#289)[^237,^](https://diagram-consortium.org/T2DGGI.html#237)[^290^](https://diagram-consortium.org/T2DGGI.html#290), Kyong-Mi Chang[^10,^](https://diagram-consortium.org/T2DGGI.html#10)[^291^](https://diagram-consortium.org/T2DGGI.html#291), Mark I McCarthy[^168,^](https://diagram-consortium.org/T2DGGI.html#168)[^292,^](https://diagram-consortium.org/T2DGGI.html#292)[^293,^](https://diagram-consortium.org/T2DGGI.html#293)[^301^](https://diagram-consortium.org/T2DGGI.html#301), James B Meigs[^163,^](https://diagram-consortium.org/T2DGGI.html#163)[^164,^](https://diagram-consortium.org/T2DGGI.html#164)[^13^](https://diagram-consortium.org/T2DGGI.html#13), Anubha Mahajan[^292,^](https://diagram-consortium.org/T2DGGI.html#292)[^168,^](https://diagram-consortium.org/T2DGGI.html#168)[^301^](https://diagram-consortium.org/T2DGGI.html#301), Cassandra N Spracklen[^294^](https://diagram-consortium.org/T2DGGI.html#294), Josep M Mercader[^13,^](https://diagram-consortium.org/T2DGGI.html#13)[^14,^](https://diagram-consortium.org/T2DGGI.html#14)[^76^](https://diagram-consortium.org/T2DGGI.html#76), Michael Boehnke[^9^](https://diagram-consortium.org/T2DGGI.html#9), Jerome I Rotter[^25^](https://diagram-consortium.org/T2DGGI.html#25), Marijana Vujkovic[^10,^](https://diagram-consortium.org/T2DGGI.html#10)[^291,^](https://diagram-consortium.org/T2DGGI.html#291)[^295^](https://diagram-consortium.org/T2DGGI.html#295), Benjamin F Voight[^10,^](https://diagram-consortium.org/T2DGGI.html#10)[^11,^](https://diagram-consortium.org/T2DGGI.html#11)[^12,^](https://diagram-consortium.org/T2DGGI.html#12)[^278^](https://diagram-consortium.org/T2DGGI.html#278), Andrew P Morris[^1,^](https://diagram-consortium.org/T2DGGI.html#1)[^4,^](https://diagram-consortium.org/T2DGGI.html#4)[^22^](https://diagram-consortium.org/T2DGGI.html#22), Eleftheria Zeggini[^4,^](https://diagram-consortium.org/T2DGGI.html#4)[^296^](https://diagram-consortium.org/T2DGGI.html#296),

1 Centre for Genetics and Genomics Versus Arthritis, Centre for Musculoskeletal Research, Division of Musculoskeletal and Dermatological Sciences, The University of Manchester, Manchester, UK
2 Department of Diabetes and Metabolic Diseases, Graduate School of Medicine, The University of Tokyo, Tokyo, Japan
3 Department of Statistical Genetics, Osaka University Graduate School of Medicine, Suita, Japan
4 Institute of Translational Genomics, Helmholtz Zentrum München, German Research Center for Environmental Health, Neuherberg, Germany
5 Center for Precision Health Research, National Human Genome Research Institute, National Institutes of Health, Bethesda, MD, USA
6 British Heart Foundation Cardiovascular Epidemiology Unit, Department of Public Health and Primary Care, University of Cambridge, Cambridge, UK
7 Heart and Lung Research Institute, University of Cambridge, Cambridge, UK
8 Department of Epidemiology, School of Public Health, Nanjing Medical University, Nanjing City, China
9 Department of Biostatistics and Center for Statistical Genetics, University of Michigan , Ann Arbor, MI, USA
10 Corporal Michael J Crescenz VA Medical Center, Philadelphia, PA, USA
11 Department of Systems Pharmacology and Translational Therapeutics, University of Pennsylvania Perelman School of Medicine, Philadelphia, PA, USA
12 Department of Genetics, University of Pennsylvania Perelman School of Medicine, Philadelphia, PA, USA
13 Programs in Metabolism and Medical and Population Genetics, Broad Institute of Harvard and MIT, Cambridge, MA, USA
14 Diabetes Unit and Center for Genomic Medicine, Massachusetts General Hospital, Boston, MA, USA
15 TIMI Study Group, Division of Cardiovascular Medicine, Brigham and Women’s Hospital, Harvard Medical School, Boston, MA, USA
16 William Harvey Research Institute, Barts and the London School of Medicine and Dentistry, Queen Mary University of London, London, UK
17 Department of Genome Informatics, Graduate School of Medicine, The University of Tokyo, Tokyo, Japan
18 Integrated Frontier Research for Medical Science Division, Institute for Open and Transdisciplinary Research Initiatives, Osaka University, Suita, Japan
19 Laboratory for Systems Genetics, RIKEN Center for Integrative Medical Sciences, Kanagawa, Japan
20 The Charles Bronfman Institute for Personalized Medicine, Icachn School of Medicine at Mount Sinai, New York, NY, USA
21 Department of Medicine, Vanderbilt University Medical Center, Nashville, TN, USA
22 Estonian Genome Centre, Institute of Genomics, University of Tartu, Tartu, Estonia
23 Nuffield Department of Population Health, University of Oxford, Oxford, UK
24 Medical Research Council Population Health Research Unit, University of Oxford, Oxford, UK
25 The Institute for Translational Genomics and Population Sciences, Department of Pediatrics, The Lundquist Institute for Biomedical Innovation (formerly Los Angeles Biomedical Research Institute) at Harbor-UCLA Medical Center, Torrance, CA, USA
26 Department of Epidemiology and Biostatistics, Imperial College London, London, UK
27 Department of Cardiology, Ealing Hosptial, London North West Healthcare NHS Trust, Middlesex, UK
28 Division of Genome Science, Department of Precision Medicine, National Institute of Health, Cheongju-si, South Korea
29 Department of Epidemiology, Gillings School of Global Public Health, University of North Carolina at Chapel Hill, Chapel Hill, NC, USA
30 Department of Gene Diagnostics and Therapeutics, Research Institute, National Center for Global Health and Medicine, Tokyo, Japan
31 Institute of Epidemiology, Helmholtz Zentrum Munchen, German Research Center for Environmental Health, Neuherberg, Germany
32 Department of Medicine, McMaster University, Hamilton, ON, Canada
33 Population Health Research Institute, Hamilton Health Sciences and McMaster University, Hamilton, ON, Canada
34 Public Health Informatics Unit, Department of Integrated Health Sciences, Nagoya University Graduate School of Medicine, Nagoya, Japan
35 MRC Epidemiology Unit, Institute of Metabolic Science, University of Cambridge School of Clinical Medicine, Cambridge, UK
36 Department of Health Data Science, University of Liverpool, Liverpool, UK
37 Division of Translational Medicine and Human Genetics, University of Pennsylvania, Philadelphia, PA, USA
38 Department of Epidemiology, Brown University School of Public Health, Providence, RI, USA
39 Department of Anthropology, University of Toronto at Mississsauga, Mississauga, ON, Canada
40 Saw Swee Hock School of Public Health, National University of Singapore and National University Health System, Singapore, Singapore
41 Department of Epidemiology, School of Public Health, University of Michigan, Ann Arbor, MI, USA
42 Center for Genomic Medicine, Kyoto University Graduate School of Medicine, Kyoto, Japan
43 deCODE Genetics, Amgen Inc., Reykjavik, Iceland
44 Novo Nordisk Foundation Center for Basic Metabolic Research, Faculty of Health and Medical Sciences, University of Copenhagen, Copenhagen, Denmark
45 Department of Biostatistics, Harvard University, Boston, MA, USA
46 Division of Sleep and Circadian Disorders, Brigham and Women's Hospital, Boston, MA, USA
47 Department of Medicine, Harvard University, Boston, MA, USA
48 Institute of Genetic Epidemiology, Department of Data Driven Medicine, Faculty of Medicine and Medical Center, University of Freiburg, Freiburg, Germany
49 Department of Epidemiology, Human Genetics, and Environmental Sciences, The University of Texas Health Science Center at Houston School of Public Health, Houston, TX, USA
50 German Center for Diabetes Research (DZD), Neuherberg, Germany
51 Research Unit of Molecular Epidemiology, Helmholtz Zentrum München, German Research Center for Environmental Health, Neuherberg, Germany
52 Department of Population and Public Health Sciences, Keck School of Medicine of USC, Los Angeles, CA, USA
53 Department of Cardiology, Leiden University Medical Center, Leiden, The Netherlands
54 Section of Gerontology and Geriatrics, Department of Internal Medicine, Leiden University Medical Center, Leiden, The Netherlands
55 Department of Internal Medicine, Seoul National University Hospital, Seoul, South Korea
56 Division of Epidemiology, Department of Medicine, Institute for Medicine and Public Health, Vanderbilt Genetics Institute, Vanderbilt University Medical Center, Nashville, TN, USA
57 Nuffield Department of Surgical Sciences, University of Oxford, Oxford, UK
58 Institute for Population and Precision Health (IPPH), Biological Sciences Division, The University of Chicago, Chicago, IL, USA
59 Department of Public Health Sciences and Center for Public Health Genomics, University of Virginia School of Medicine, Charlottesville, VA, USA
60 Genomic Research on Complex Diseases (GRC-Group), CSIR-Centre for Cellular and Molecular Biology (CSIR-CCMB), Hyderabad, India
61 Department of Medicine and Therapeutics, The Chinese University of Hong Kong, Hong Kong, China
62 Chinese University of Hong Kong-Shanghai Jiao Tong University Joint Research Centre in Diabetes Genomics and Precision Medicine, The Chinese University of Hong Kong, Hong Kong, China
63 Samsung Advanced Institute for Health Sciences & Technology (SAIHST), Sungkyunkwan University, Samsung Medical Center, Seoul, South Korea
64 Department of Preventive Medicine, Northwestern University Feinberg School of Medicine , Chicago, IL, USA
65 Institute of Biomedical Sciences, Academia Sinica, Taipei, Taiwan
66 Department of Genetics, University of North Carolina at Chapel Hill, Chapel Hill, NC, USA
67 Department of Human Genetics, Wellcome Sanger Institute, Wellcome Genome Campus, Hinxton, UK
68 Laboratory of Neurogenetics, National Institute on Aging, National Institutes of Health, Bethesda, MD, USA
69 Department of Medicine, Johns Hopkins University School of Medicine, Baltimore, MD, USA
70 Center for Research on Genomics and Global Health, National Human Genome Research Institute, National Institutes of Health, Bethesda, MD, USA
71 Cardiovascular Health Research Unit, Department of Medicine, University of Washington, Seattle, WA, USA
72 Division of Academics, Ochsner Health, New Orleans, LA, USA
73 Division of Statistical Genomics, Washington University School of Medicine, St. Louis, MO, USA
74 Department of Research & Evaluation, Division of Biostatistics Research, Kaiser Permanente of Southern California, Pasadena, CA, USA
75 Department of Biomedical Science, Hallym University, Chuncheon, South Korea
76 Harvard Medical School, Boston, MA, USA
77 Metabolic Research Laboratories, Wellcome Trust-Medical Research Counci Institute of Metabolic Science, Department of Clinical Biochemistry, University of Cambridge, Cambridge, UK
78 Department of Epidemiology, University of Groningen, University Medical Centre Groningen, Groningen, The Netherlands
79 Department of Bioinformatics, Isfahan University of Medical Sciences, Isfahan, Iran
80 Department of Medicine, Division of Endocrinology, Diabetes and Metabolism, Cedars-Sinai Medical Center, Los Angeles, CA, USA
81 Lund University Diabetes Centre, Department of Clinical Sciences, Lund University, Skåne University Hospital, Malmö, Sweden
82 Department of Nutrition, Gillings School of Global Public Health, University of North Carolina at Chapel Hill, Chapel Hill, NC, USA
83 Unidad de Investigación en Enfermedades Metabólicas and Departamento de Endocrinología y Metabolismo., Instituto Nacional de Ciencias Médicas y Nutrición Salvador Zubirán. , Mexico City, Mexico
84 Steno Diabetes Center Copenhagen, Herlev, Denmark
85 The Bioinformatics Center, Department of Biology, University of Copenhagen, Copenhagen, Denmark
86 Department of Health Research Methods, Evidence, and Impact, McMaster University, Hamilton, ON, Canada
87 Department of Epidemiology and Prevention, Division of Public Health Sciences, Wake Forest School of Medicine, Winston-Salem, NC, USA
88 Institute of Regional Health Research, University of Southern Denmark, Odense, Denmark
89 Department of Clinical Biochemistry, Vejle Hospital, Vejle, Denmark
90 Department of Medicine, Division of Endocrinology and Diabetes, Keck School of Medicine of USC, Los Angeles, CA, USA
91 Department of Internal Medicine, University of Michigan, Ann Arbor, MI, USA
92 British Heart Foundation Centre of Research Excellence, School of Clinical Medicine, Addenbrooke's Hospital, University of Cambridge, Cambridge, UK
93 Health Data Research UK Cambridge, Wellcome Genome Campus and University of Cambridge, Hinxton, UK
94 National Institute for Health and Care Research (NIHR) Blood and Transplant Unit (BTRU) in Donor Health and Behaviour, Heart and Lung Research Institute, University of Cambridge, Cambridge, UK
95 Inserm U1283, CNRS UMR 8199, European Genomic Institute for Diabetes (EGID), Institut Pasteur de Lille, Lille University Hospital, Lille, France
96 University of Lille, Lille, France
97 Li Ka Shing Institute of Health Sciences, The Chinese University of Hong Kong, Hong Kong, China
98 Hong Kong Institute of Diabetes and Obesity, The Chinese University of Hong Kong, Hong Kong, China
99 Singapore Eye Research Institute, Singapore National Eye Centre, Singapore, Singapore
100 Exeter Centre of Excellence in Diabetes (ExCEeD), Exeter Medical School, University of Exeter, Exeter, UK
101 Wellcome Sanger Institute, Wellcome Genome Campus, Hinxton, UK
102 Department of Biostatistics and Data Science, Wake Forest School of Medicine, Winston-Salem, NC, USA
103 Division of Endocrinology and Metabolism, Department of Internal Medicine, National Taiwan University Hospital, Taipei, Taiwan
104 Institute of Epidemiology and Preventive Medicine, National Taiwan University, Taipei, Taiwan
105 Department of Medicine, University of Vermont, Colchester, VT, USA
106 Section on Endocrinology and Metabolism, Department of Internal Medicine, Wake Forest School of Medicine, Winston-Salem, NC, USA
107 Department of Medicine, Faculty of Medicine, University of Kelaniya, Ragama, Sri Lanka
108 Department of Nutrition and Dietetics, Harokopio University of Athens, Athens, Greece
109 Center for Genomics and Personalized Medicine Research, Wake Forest School of Medicine, Winston-Salem, NC, USA
110 Carolina Population Center, University of North Carolina at Chapel Hill, Chapel Hill, NC, USA
111 Department of Nephrology and Medical Intensive Care Medicine, Charité Universitätsmedizin Berlin, Berlin, Germany
112 Department of Nephrology and Hypertension, Friedrich-Alexander-Universität Erlangen-Nürnberg, Erlangen, Germany
113 Department of Biostatistics, University of Washington, Seattle, WA, USA
114 California Pacific Medical Center Research Institute, San Francisco, CA, USA
115 Laboratory of Epidemiology and Population Sciences, National Institute on Aging, National Institutes of Health, Baltimore, MD, USA
116 Institute of Mathematics and Statistics, University of Tartu, Tartu, Estonia
117 Robertson Centre for Biostatistics, University of Glasgow, Glasgow, UK
118 Department of Epidemiology, Erasmus MC University Medical Center, Rotterdam, The Netherlands
119 Genetics of Complex Traits, University of Exeter Medical School, University of Exeter, Exeter, UK
120 Department of Internal Medicine, Wake Forest School of Medicine, Winston-Salem, NC, USA
121 Department of Medicine, Division of Endocrinology and Metabolism, Lundquist Research Institute at Harbor-UCLA Medical Center, Torrance, CA, USA
122 Department of Public Health and Caring Sciences, Uppsala University, Uppsala, Sweden
123 Centro de Estudios en Diabetes, Unidad de Investigacion en Diabetes y Riesgo Cardiovascular, Centro de Investigacion en Salud Poblacional, Instituto Nacional de Salud Publica, Mexico City, Mexico
124 Department of Laboratory Medicine and Pathology, University of Minnesota, Minneapolis, MN, USA
125 Genomics and Computational Biology Graduate Group, University of Pennsylvania Perelman School of Medicine, Philadelphia, PA, USA
126 Institute for Molecular Medicine Finland (FIMM), University of Helsinki, Helsinki, Finland
127 Folkhalsan Research Center, Helsinki, Finland
128 University of Exeter Medical School, University of Exeter, Exeter, UK
129 Institute for Clinical Diabetology, German Diabetes Center, Leibniz Center for Diabetes Research at Heinrich Heine University Düsseldorf, Düsseldorf, Germany
130 Department of Endocrinology and Diabetology, Medical Faculty and University Hospital Düsseldorf, Heinrich Heine University Düsseldorf, Düsseldorf, Germany
131 Laboratory for Genomics of Diabetes and Metabolism, RIKEN Center for Integrative Medical Sciences, Kanagawa, Japan
132 Department of Biostatistics, Gillings School of Global Public Health, University of North Carolina at Chapel Hill, Chapel Hill, NC, USA
133 Department of Internal Medicine, Diabetes and Metabolism Research Center, The Ohio State University Wexner Medical Center, Columbus, OH, USA
134 Center for Global Cardiometabolic Health, Brown University, Providence, RI, USA
135 Shanghai-MOST Key Laboratory of Health and Disease Genomics, Shanghai Institute for Biomedical and Pharmaceutical Technologies, Shanghai, China
136 Division of Endocrine and Metabolism, Tri-Service General Hospital Songshan Branch, Taipei, Taiwan
137 School of Medicine, National Defense Medical Center, Taipei, Taiwan
138 Division of Genome Science, Department of Precision Medicine, National Institute of Health, Cheongju-si, Korea
139 Section of Endocrinology and Metabolism, Department of Medicine, Taipei Veterans General Hospital, Taipei, Taiwan
140 School of Medicine, National Yang Ming Chiao Tung University, Taipei, Taiwan
141 Department of Environmental and Preventive Medicine, Jichi Medical University School of Medicine, Shimotsuke, Japan
142 University of Chicago Research Bangladesh, Dhaka, Bangladesh
143 Institute of Molecular and Clinical Ophthalmology Basel, Basel, Switzerland
144 Center for Clinical Research and Prevention, Bispebjerg and Frederiksberg Hospital, Frederiksberg, Denmark
145 Faculty of Health and Medical Sciences, University of Copenhagen, Copenhagen, Denmark
146 Faculty of Medicine, Aalborg University, Aalborg, Denmark
147 Department of Clinical Diabetes, Endocrinology and Metabolism, Department of Translational Research and Cellular Therapeutics, City of Hope, Duarte, CA, USA
148 Department of Public Health, Faculty of Medicine, University of Kelaniya, Ragama, Sri Lanka
149 Department of Clinical Gene Therapy, Osaka University Graduate School of Medicine, Osaka, Japan
150 Department of Geriatric and General Medicine, Graduate School of Medicine, Osaka University, Osaka, Japan
151 Division of General Internal Medicine and Geriatrics, Department of Medicine, Northwestern University Feinberg School of Medicine, Chicago, IL, USA
152 Center for Health Information Partnerships, Institute for Public Health and Medicine, Northwestern University Feinberg School of Medicine, Chicago, IL, USA
153 Genome Institute of Singapore, Agency for Science, Technology and Research, Singapore, Singapore
154 Department of Molecular Cell Biology, Sungkyunkwan University School of Medicine, Suwon, South Korea
155 Institute of Genetic Epidemiology, Medical University of Innsbruck, Innsbruck, Austria
156 Institute of Clinical Medicine, Internal Medicine, University of Eastern Finland and Kuopio University Hospital, Kuopio, Finland
157 Department of Medicine, University of Colorado Denver, Anschutz Medical Campus, Aurora, CO, USA
158 VA Salt Lake City Health Care System, Salt Lake City, UT, USA
159 Department of Internal Medicine, University of Utah School of Medicine, Salt Lake City, UT, USA
160 Soochunhyang Institute of Medi-bio Science and Division of Endocrinology, Department of Internal Medicine, Soochunhyang University College of Medicine, Cheonan, South Korea
161 Department of Medicine, Samsung Medical Center, Sungkyunkwan University School of Medicine, Seoul, South Korea
162 USC-Office of Population Studies Foundation Inc., University of San Carlos, Cebu City, Philippines
163 Department of Medicine, Harvard Medical School, Boston, MA, USA
164 Division of General Internal Medicine, Massachusetts General Hospital, Boston, MA, USA
165 Department of Epidemiology and Biostatistics, School of Public Health, Peking University, Beijing, China
166 Peking University Center for Public Health and Epidemic Preparedness and Response, Beijing, China
167 Department of Clinical Epidemiology, Leiden University Medical Center, Leiden, The Netherlands
168 Wellcome Centre for Human Genetics, Nuffield Department of Medicine, University of Oxford, Oxford, UK
169 Program in Medical and Population Genetics, Broad Institute, Cambridge, MA, USA
170 Big Data Institute, Li Ka Shing Centre For Health Information and Discovery, University of Oxford, Oxford, UK
171 Department of Clinical Medicine, Faculty of Health and Medical Sciences, University of Copenhagen, Copenhagen, Denmark
172 Department of Biostatistics, Boston University School of Public Health, Boston, MA, USA
173 Department of Medicine, Yong Loo Lin School of Medicine, National University of Singapore and National University Health System, Singapore, Singapore
174 McDonnell Genome Institute, Washington University School of Medicine, St. Louis, MO, USA
175 Department of Medicine, Division of Genomics and Bioinformatics, Washington University School of Medicine, St. Louis, MO, USA
176 Department of Biostatistics and Data Science, The University of Texas Health Science Center at Houston School of Public Health, Houston, TX, USA
177 Department of Clinical Sciences, Diabetes and Endocrinology, Lund University Diabetes Centre, Malmö, Sweden
178 Department of Clinical Science, Center for Diabetes Research, University of Bergen, Bergen, Norway
179 Department of Advanced Genomic and Laboratory Medicine, Graduate School of Medicine, University of the Ryukyus, Okinawa, Japan
180 Division of Clinical Laboratory and Blood Transfusion, University of the Ryukyus Hospital, Okinawa, Japan
181 Dromokaiteio Psychiatric Hospital, National and Kapodistrian University of Athens, Athens, Greece
182 Computational Biology and Medical Sciences, Graduate School of Frontier Sciences, The University of Tokyo, Tokyo, Japan
183 Institute of Human Genetics, Helmholtz Zentrum München, German Research Center for Environmental Health, Neuherberg, Germany
184 Institute of Human Genetics, Technical University Munich, Munich, Germany
185 German Centre for Cardiovascular Research (DZHK), Partner Site Munich Heart Alliance, Munich, Germany
186 The Usher Institute to the Population Health Sciences and Informatics, University of Edinburgh, Edinburgh, UK
187 Department of Medicine and Pharmacology, New York Medical College, Valhalla, NY, USA
188 Data Tecnica International LLC, Glen Echo, MD, USA
189 Center for Alzheimer's and Related Dementias, National Institutes of Health, Bethesda, MD, USA
190 Laboratory of Statistical Immunology, Immunology Frontier Research Center (WPI-IFReC), Osaka University, Suita, Japan
191 Premium Research Institute for Human Metaverse Medicine (WPI-PRIMe), Osaka University, Suita, Japan
192 Instituto Nacional de Medicina Genómica, Mexico City, Mexico
193 Division of Pulmonary, Allergy, and Critical Care Medicine, Department of Medicine, University of Pittsburgh, Pittsburgh, PA, USA
194 Division of Epidemiology and Community Health, School of Public Health, University of Minnesota, Minneapolis, MN, USA
195 Institute for Medical Information Processing, Biometry and Epidemiology, Ludwig-Maximilians-Universität München, Munich, Germany
196 Department of Diabetes and Endocrinology, Nelson R Mandela School of Medicine, College of Health Sciences, University of KwaZulu-Natal, Durban, South Africa
197 Academy of Scientific and Innovative Research, CSIR-Human Resource Development Campus, Ghaziabad, India
198 Genomics and Molecular Medicine Unit, CSIR-Institute of Genomics and Integrative Biology, New Delhi, India
199 Department of Preventive Medicine, Northwestern University Feinberg School of Medicine, Chicago, IL, USA
200 Fred Hutchinson Cancer Research Center, Seattle, WA, USA
201 Ophthalmology and Visual Sciences Academic Clinical Program (Eye ACP), Duke-NUS Medical School, Singapore, Singapore
202 Department of Ophthalmology, Yong Loo Lin School of Medicine, National University of Singapore and National University Health System, Singapore, Singapore
203 School of Cardiovascular and Metabolic Health, University of Glasgow, Glasgow, UK
204 Survey Research Center, Institute for Social Research, University of Michigan, Ann Arbor, MI, USA
205 Institute of Genetic Epidemiology, Helmholtz Zentrum Munchen, German Research Center for Environmental Health, Neuherberg, Germany
206 Institute for Medical Biostatistics, Epidemiology and Informatics (IMBEI), University Medical Center, JohannesGutenberg University, Mainz, Germany
207 Chair of Genetic Epidemiology, Institute of Medical Information Processing, Biometry, and Epidemiology, Faculty of Medicine, Ludwig-Maximilians-Universität München, Munich, Germany
208 Faculty of Medicine, University of Iceland, Reykjavik, Iceland
209 Faculty of Medicine, Macau University of Science and Technology, Macau, China
210 Department of Medical Genetics and Medical Research, China Medical University Hospital, Taichung, Taiwan
211 Population Health Unit, Finnish Institute for Health and Welfare, Helsinki, Finland, Finnish Institute for Health and Welfare, Helsinki, Finland
212 National School of Public Health, Madrid, Spain
213 Department of Public Health, University of Helsinki, Helsinki, Finland
214 Diabetes Research Group, King Abdulaziz University, Jeddah, Saudi Arabia
215 Unidad de Biología Molecular y Medicina Genómica, Instituto Nacional de Ciencias Médicas y Nutrición Salvador Zubirán, Mexico City, Mexico
216 Departamento de Medicina Genómica y Toxiología Ambiental, Instituto de Investigaciones Biomédicas, UNAM, Mexico City, Mexico
217 Unidad de Investigacion Medica en Bioquimica, Hospital de Especialidades, Centro Medico Nacional Siglo XXI, Instituto Mexicano del Seguro Social, Mexico City, Mexico
218 Einthoven Laboratory for Experimental Vascular Medicine, Leiden University Medical Center, Leiden, The Netherlands
219 Department of Human Genetics, Leiden University Medical Center, Leiden, The Netherlands
220 Department of Clinical Chemistry, Laboratory of Genetic Metabolic Disease, Amsterdam University Medical Center, Amsterdam, The Netherlands
221 Southern California Eye Institute, CHA Hollywood Presbyterian Hospital, Los Angeles, CA, USA
222 Unidad de Investigación Médica en Epidemiologia Clinica, Hospital de Especialidades, Centro Medico Nacional Siglo XXI, Instituto Mexicano del Seguro Social, Mexico City, Mexico
223 Department of Internal Medicine, Division of Endocrinology, Leiden University Medical Center, Leiden, The Netherlands
224 Department of Public Health, Aarhus University, Aarhus, Denmark
225 Danish Diabetes Academy, Odense, Denmark
226 Diabetology Research Centre, King Edward Memorial Hospital and Research Centre, Pune, India
227 Department of Medical Biochemistry, Kurume University School of Medicine, Kurume, Japan
228 Department of Pediatrics, Osaka University Graduate School of Medicine, Suita, Japan
229 Division of Cancer Control and Population Sciences, UPMC Hillman Cancer Center, University of Pittsburgh, Pittsburgh, PA, USA
230 Department of Epidemiology, Graduate School of Public Health, University of Pittsburgh, Pittsburgh, PA, USA
231 Department of Pediatrics, Division of Genetic and Genomic Medicine, UCI Irvine School of Medicine, Irvine, CA, USA
232 Department of Anti-Aging Medicine, Ehime University Graduate School of Medicine, Touon, Japan
233 Division of Pulmonary, Critical Care, and Sleep Medicine, Beth Israel Deaconess Medical Center, Boston, MA, USA
234 Department of Medical Sciences, Uppsala University, Uppsala, Sweden
235 Institute of Molecular Medicine, The University of Texas Health Science Center at Houston School of Public Health, Houston, TX, USA
236 Human Genetics Center, University of Texas Health Science Center at Houston, Houston, TX, US
237 Department of Medicine, Stanford University School of Medicine, Stanford, CA, USA
238 Department of Medical Sciences, Molecular Epidemiology and Science for Life Laboratory, Uppsala University, Uppsala, Sweden
239 Department of Epidemiology, University of Washington, Seattle, WA, USA
240 Department of Health Systems and Population Health, University of Washington, Seattle, WA, USA
241 Beijing Institute of Ophthalmology, Ophthalmology and Visual Sciences Key Laboratory, Beijing Tongren Hospital, Capital Medical University, Beijing, China
242 Department of Medicine, Division of Cardiology, Duke University School of Medicine, Durham, NC, USA
243 Kurume University School of Medicine, Kurume, Japan
244 Department of Medicine, McGill University, Montreal, QC, Canada
245 Department of Human Genetics, McGill University, Montreal, QC, Canada
246 Department of Metabolism, Digestion and Reproduction, Imperial College London, London, UK
247 Division of Cardiovascular Medicine, Beth Israel Deaconess Medical Center, Boston, MA, USA
248 Division of Endocrinology and Metabolism, Department of Medicine, Taichung Veterans General Hospital, Taichung, Taiwan
249 Division of Endocrinology, Metabolism, and Molecular Medicine, Department of Medicine, Northwestern University Feinberg School of Medicine, Chicago, IL, USA
250 Center for Genetic Medicine, Northwestern University Feinberg School of Medicine, Chicago, IL, USA
251 Department of Anthropology, Northwestern University, Evanston, IL, USA
252 Department of Endocrinology, Helsinki University Hospital, Helsinki, Finland
253 Systems Genomics Laboratory, School of Biotechnology, Jawaharlal Nehru University, New Delhi, India
254 Department of Pathology and Molecular Medicine, McMaster University, Hamilton, ON, Canada
255 Department of Molecular Medicine and Biopharmaceutical Sciences, Graduate School of Convergence Science and Technology, Seoul National University, Seoul, South Korea
256 Netherlands Heart Institute, Utrecht, The Netherlands
257 Center for Public Health Genomics, University of Virginia School of Medicine, Charlottesville, VA, USA
258 Research Unit of Molecular Epidemiology, Helmholtz Zentrum München, German Research Center for Environmental Health, Munich, Germany
259 Duke-NUS Medical School, Singapore, Singapore
260 Department of Epidemiology, Biostatistics and Occupational Health, McGill University, Montreal, QC, Canada
261 Singapore Institute for Clinical Sciences, Agency for Science Technology and Research (A*STAR), Singapore, Singapore
262 Healthy Longevity Translational Research Programme, Yong Loo Lin School of Medicine, National University of Singapore, Singapore, Singapore
263 Center for Diabetes Research, Wake Forest School of Medicine, Winston-Salem, NC, USA
264 Department of Biochemistry, Wake Forest School of Medicine, Winston-Salem, NC, USA
265 Pat Macpherson Centre for Pharmacogenetics and Pharmacogenomics, University of Dundee, Dundee, UK
266 Imperial College Healthcare NHS Trust, Imperial College London, London, UK
267 MRC-PHE Centre for Environment and Health, Imperial College London, London, UK
268 National Heart and Lung Institute, Imperial College London, London, UK
269 Department of Medicine, Brown University Alpert School of Medicine, Providence, RI, USA
270 Department of Medicine, Columbia University Irving Medical Center, New York, NY, USA
271 Department of Cardiology, Columbia University Irving Medical Center, New York, NY, USA
272 Center for Non-Communicable Diseases, Karachi, Pakistan
273 Computational Medicine, Berlin Institute of Health at Charité–Universitätsmedizin, Berlin, Germany
274 Precision Healthcare University Research Institute, Queen Mary University of London, London, UK
275 Department of Preventive Medicine, Keck School of Medicine of USC, Los Angeles, CA, USA
276 The Mindich Child Health and Development Institute, Ichan School of Medicine at Mount Sinai, New York, NY, USA
277 Division of Translational Medicine and Therapeutics, Department of Medicine, University of Pennsylvania Perelman School of Medicine, Philadelphia, PA, USA
278 Institute for Translational Medicine and Therapeutics, University of Pennsylvania Perelman School of Medicine, Philadelphia, PA, USA
279 Department of Pediatrics, University of Pennsylvania Perelman School of Medicine, Philadelphia, PA, USA
280 Center for Precision Medicine, University of Pennsylvania - Perelman School of Medicine, Philadelphia, PA, USA
281 Institute for Biomedical Informatics, University of Pennsylvania Perelman School of Medicine, Philadelphia, PA, USA
282 Department of Psychiatry, University of Michigan, Ann Arbor, MI, USA
283 Blizard Institute, Queen Mary University of London, London, UK
284 Institute for Population Health Sciences, Barts and the London School of Medicine and Dentistry, Queen Mary University of London, London, UK
285 All of Us Research Program, National Institutes of Health, Bethesda, MD, USA
286 Toranomon Hospital, Tokyo, Japan
287 Lee Kong Chian School of Medicine, Nanyang Technological University, Singapore, Singapore
288 Vanderbilt Genetics Institute, Division of Genetic Medicine, Vanderbilt University Medical Center, Nashville, TN, USA
289 VA Palo Alto Health Care System, Palo Alto, CA, USA
290 Stanford Cardiovascular Institute, Stanford University School of Medicine, Stanford, CA, USA
291 Department of Medicine, University of Pennsylvania Perelman School of Medicine, Philadelphia, PA, USA
292 Oxford Centre for Diabetes, Endocrinology and Metabolism, Radcliffe Department of Medicine, University of Oxford, Oxford, UK
293 Oxford NIHR Biomedical Research Centre, Churchill Hosptial, Oxford University Hospitals NHS Foundation Trust, Oxford, UK
294 Department of Biostatistics and Epidemiology, University of Massachusetts Amherst, Amherst, MA, USA
295 Department of Biostatistics, Epidemiology and Informatics, University of Pennsylvania Perelman School of Medicine, Philadelphia, PA, USA
296 TUM School of Medicine and Health, Technical University of Munich and Klinikum Rechts der Isar, Munich, Germany
297 Graduate School of Experimental Medicine, Technical University of Munich, Munich, Germany
298 Munich School for Data Science, Helmholtz Munich, Neuherberg, Germany
299 Academy of Scientific and Innovative Research (AcSIR), Ghaziabad, India
300 Science and Engineering Research Board (SERB), Department of Science and Technology, Ministry of Science and Technology, Government of India, New Delhi, India

Present Address:
301 Genentech, South San Francisco, CA, USA
302 Regeneron Genetics Center, Tarrytown, NY, USA
